# Supplementary material for: Undescribed Cyclohexene and Benzofuran Alkenyl Derivatives from Choerospondias axillaris, a Potential Hypoglycemic Fruit
Source: Foods. 2024 May 11;13(10):1495. doi: 10.3390/foods13101495 (PMC11119685; doi:10.3390/foods13101495)

Undescribed Cyclohexene and Benzofuran Alkenyl Derivatives from *Choerospondias axillaris*, a Potential Hypoglycemic Fruit

Ermias Tamiru Weldetsadik [1,2], Na Li [1], Jingjuan Li [1,2], Jiahuan Shang [1], Hongtao Zhu [1], and Yingjun Zhang [1,3,*]

1 State Key Laboratory of Phytochemistry and Plant Resources of West China, Kunming Institute of Botany, Chinese Academy of Sciences, Kunming 650201, China

2 University of Chinese Academy of Sciences, Beijing 100049, China

3 Yunnan Key Laboratory of Natural Medicinal Chemistry, Kunming Institute of Botany, Chinese Academy of Sciences, Kunming 650201, China

* Correspondence: zhangyj@mail.kib.ac.cn (Y.Z.); Tel.: + 86 871 6522 3235

* Corresponding author.

E-mail address: zhangyj@mail.kib.ac.cn (Y.J. Zhang).

Contents of Supplementary Data

Figure S1.1. ^{1}H NMR spectrum of compound **1** in CD_3OD. 4
Figure S1.2. ^{13}C NMR and DEPT spectrum of compound **1** in CD_3OD. 4
Figure S1.3. ^{1}H-^{1}H COSY spectrum of compound **1** in CD_3OD. 5
Figure S1.4. HSQC spectrum of compound **1** in CD_3OD. 5
Figure S1.5. HMBC spectrum of compound **1** in CD_3OD. 6
Figure S1.6. ROESY spectrum of compound **1** in CD_3OD. 6
Figure S1.7. HRESIMS spectrum of compound **1**. 7
Figure S1.8. UV spectrum of compound **1** in MeOH. 8
Figure S1.9. IR spectrum of compound **1** in KBr disk. 8
Figure S2.1. ^{1}H NMR spectrum of compound **2** in CD_3OD. 9
Figure S2.2. ^{13}C NMR and DEPT spectrum of compound **2** in CD_3OD. 9
Figure S2.3. ^{1}H-^{1}H COSY spectrum of compound **2** in CD_3OD. 10
Figure S2.4. HSQC spectrum of compound **2** in CD_3OD. 10
Figure S2.5. HMBC spectrum of compound **2** in CD_3OD. 11
Figure S2.6. ROESY spectrum of compound **2** in CD_3OD. 11
Figure S2.7. HRESIMS spectrum of compound **2**. 12
Figure S2.8. UV spectrum of compound **2** in MeOH. 13
Figure S2.9. IR spectrum of compound **2** in KBr disk. 13
Figure S3.1. ^{1}H NMR spectrum of compound **3** in CD_3OD. 14
Figure S3.2. ^{13}C NMR and DEPT spectrum of compound **3** in CD_3COCD_3. 14
Figure S3.3. ^{1}H-^{1}H COSY spectrum of compound **3** in CD_3OD. 15
Figure S3.4. HSQC spectrum of compound **3** in CD_3OD. 15
Figure S3.5. HMBC spectrum of compound **3** in CD_3OD. 16
Figure S3.6. ROESY spectrum of compound **3** in CD_3OD. 16
Figure S3.7. HRESIMS Spectrum of compound **3**. 17
Figure S3.8. UV Spectrum of compound **3** in MeOH. 18
Figure S3.9. IR Spectrum of compound **3** in KBr disk. 18
Figure S4.1. ^{1}H NMR spectrum of compound **4** in CD_3OD. 19
Figure S4.2. ^{13}C NMR and DEPT spectrum of compound **4** in CD_3OD. 19
Figure S4.3. ^{1}H-^{1}H COSY spectrum of compound **4** in CD_3OD. 20
Figure S4.4. HSQC spectrum of compound **4** in CD_3OD. 20
Figure S4.5. HMBC spectrum of compound **4** in CD_3OD. 21
Figure S4.6. ROESY spectrum of compound **4** in CD_3OD. 21
Figure S4.7. HRESIMS spectrum of compound **4**. 22

Figure S4.8. UV spectrum of compound **4** in MeOH. 23
Figure S4.9. IR spectrum of compound **4** in KBr disk. 23
Figure S5.1. ^{1}H NMR spectrum of compound **5** in CD_3OD. 24
Figure S5.2. ^{13}C NMR and DEPT spectrum of compound **5** in CD_3OD. 24
Figure S5.3. ^{1}H-^{1}H COSY spectrum of compound **5** in CD_3OD. 25
Figure S5.4. HSQC spectrum of compound **5** in CD_3OD. 25
Figure S5.5. HMBC spectrum of compound **5** in CD_3OD. 26
Figure S5.6. ROESY spectrum of compound **5** in CD_3OD. 26
Figure S5.7. HRESIMS spectrum of compound **5**. 27
Figure S5.8. UV spectrum of compound **5** in MeOH. 28
Figure S5.9. IR spectrum of compound **5** in KBr disk. 28
Figure S6.1. ^{1}H NMR spectrum of compound **6** in CD_3OD. 29
Figure S6.2. ^{13}C NMR and DEPT spectrum of compound **6** in CD_3OD. 29
Figure S6.3. ^{1}H-^{1}H COSY spectrum of compound **6** in CD_3OD. 30
Figure S6.4. HSQC spectrum of compound **6** in CD_3OD. 30
Figure S6.5. HMBC spectrum of compound **6** in CD_3OD. 31
Figure S6.6. HRESIMS spectrum of compound **6**. 32
Figure S6.7. UV spectrum of compound **6** in MeOH. 33
Figure S6.8. IR spectrum of compound **6** in KBr disk. 33
Figure S7.1. ^{1}H NMR spectrum of compound **7** in CD_3OD. 34
Figure S7.2. ^{13}C NMR and DEPT spectrum of compound **7** in CD_3OD. 34
Figure S7.3. ^{1}H-^{1}H COSY spectrum of compound **7** in CD_3OD. 35
Figure S7.4. HSQC spectrum of compound **7** in CD_3OD. 35
Figure S7.5. HMBC spectrum of compound **7** in CD_3OD. 36
Figure S7.6. HRESIMS spectrum of compound **7**. 37
Figure S7.7. UV spectrum of compound **7** in MeOH. 38
Figure S7.8. IR spectrum of compound **7** in KBr disk. 38
Figure S8.1. ^{1}H NMR spectrum of compound **8** in CD_3OD. 39
Figure S8.2. ^{13}C NMR and DEPT spectrum of compound **8** in CD_3OD. 39
Figure S8.3. HSQC spectrum of compound **8** in CD_3OD. 40
Figure S8.4. HMBC spectrum of compound **8** in CD_3OD. 40

Figure S1.1. ^{1}H NMR spectrum of compound **1** in CD_3OD.

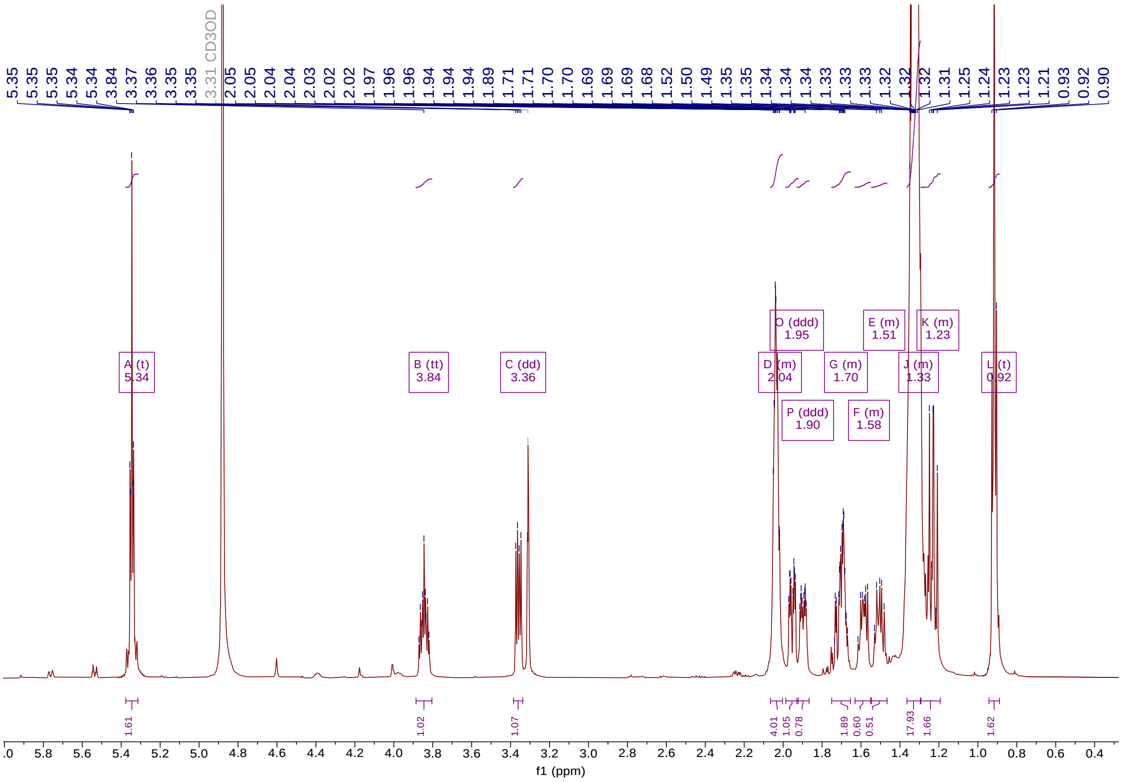

Figure S1.2. ^{13}C NMR and DEPT spectrum of compound **1** in CD_3OD.

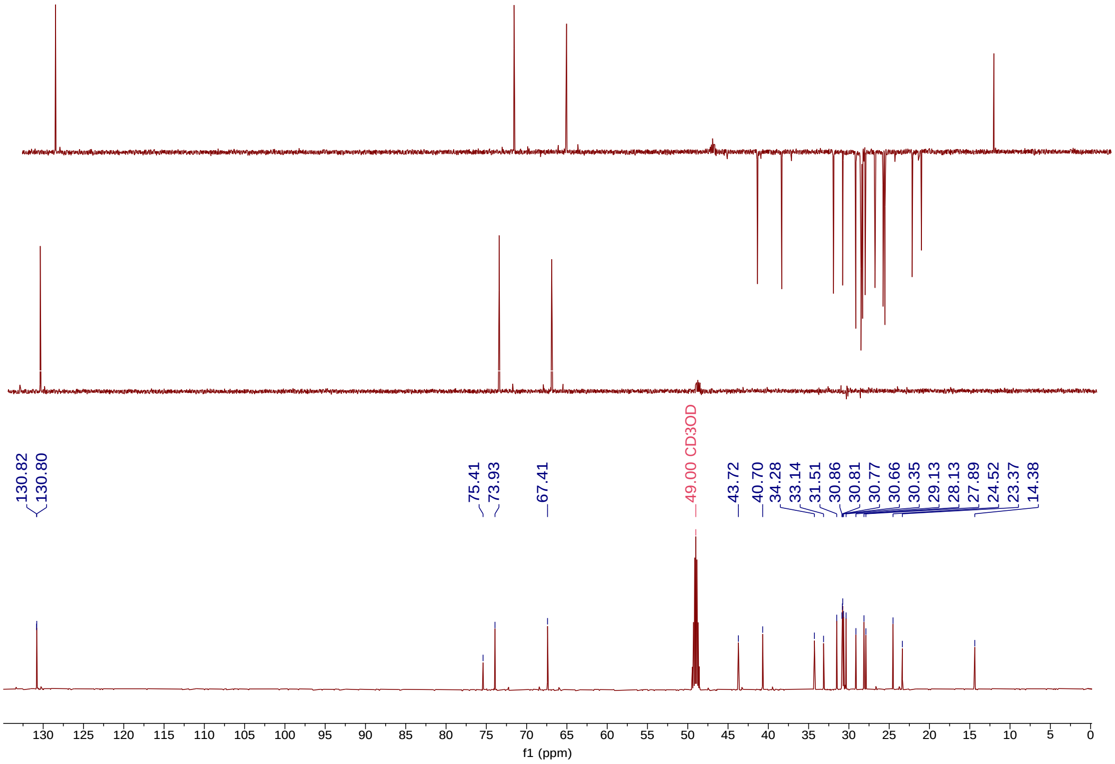

Figure S1.3. ^{1}H-^{1}H COSY spectrum of compound **1** in CD_3OD.

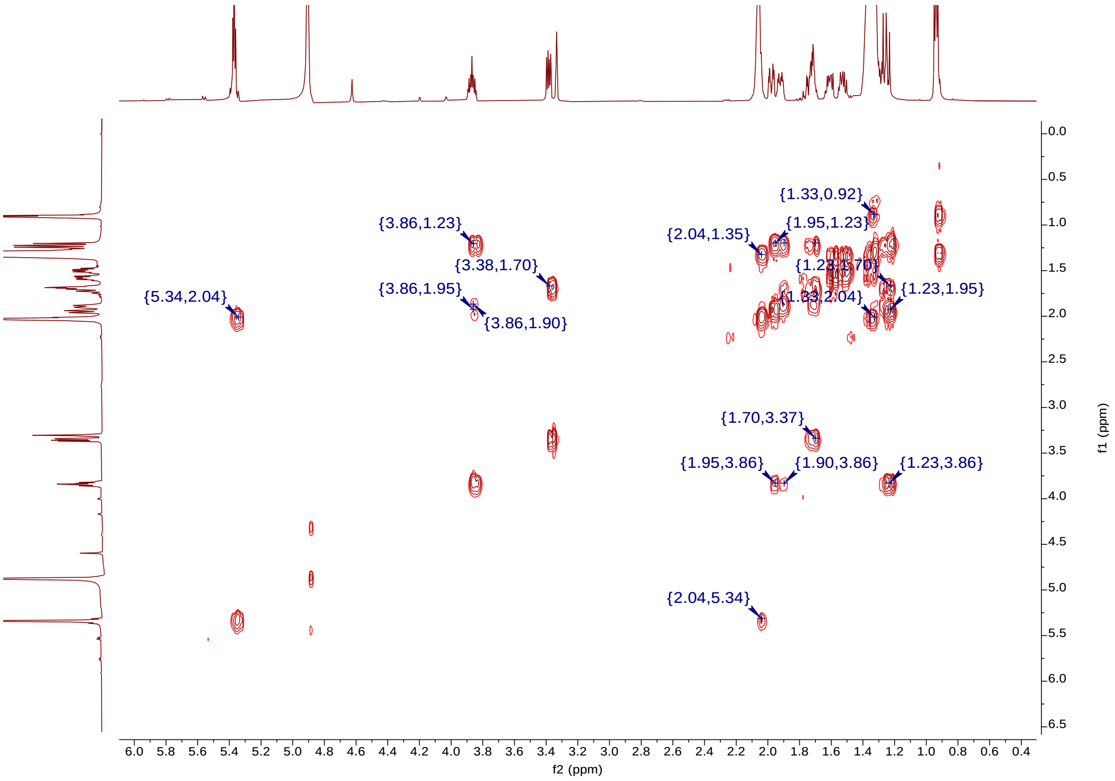

Figure S1.4. HSQC spectrum of compound **1** in CD_3OD.

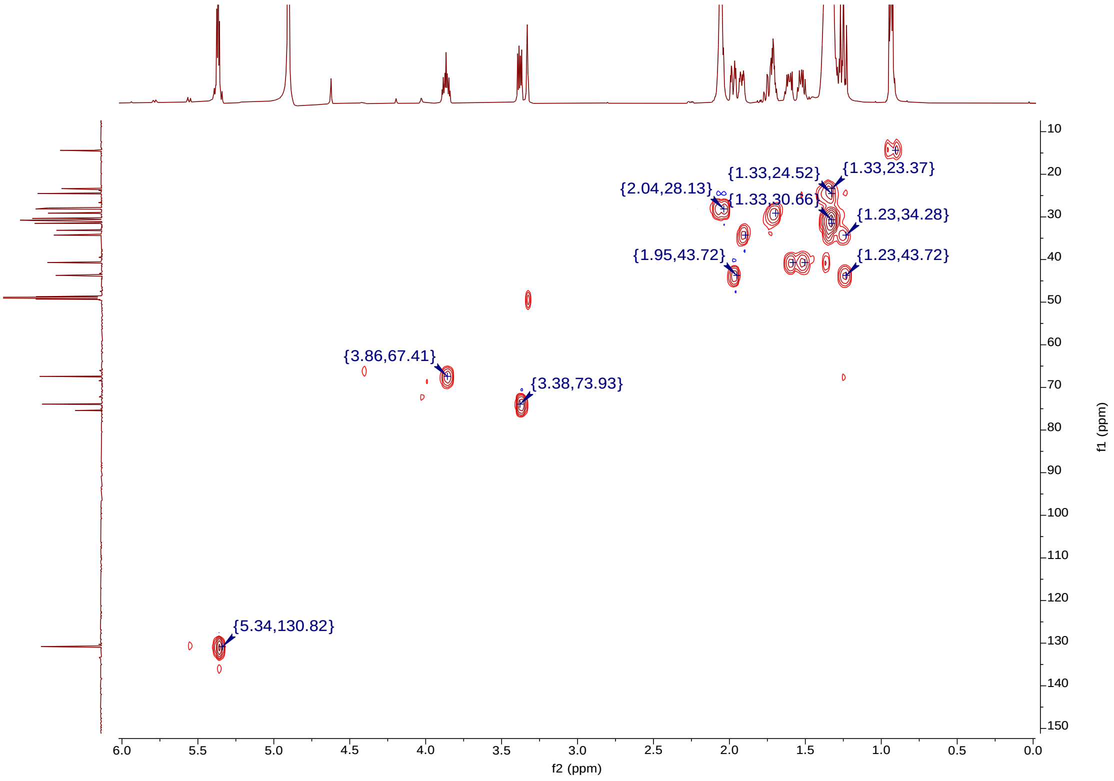

Figure S1.5. HMBC spectrum of compound **1** in CD_3OD.

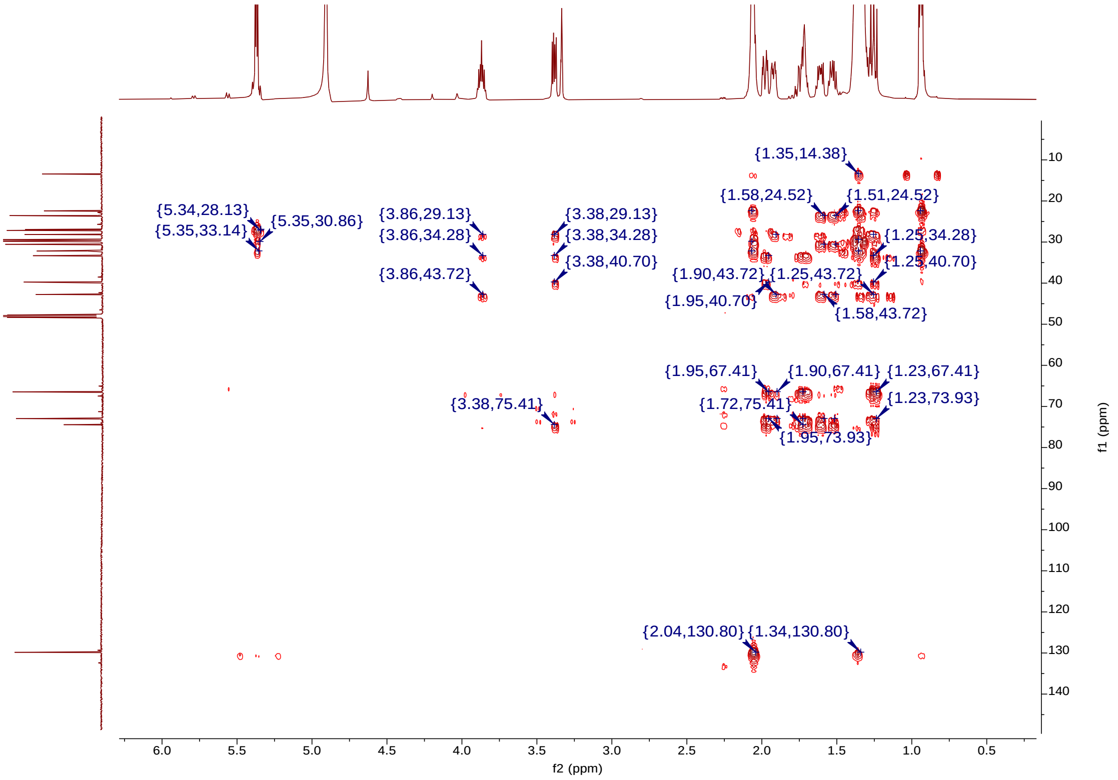

Figure S1.6. ROESY spectrum of compound **1** in CD_3OD.

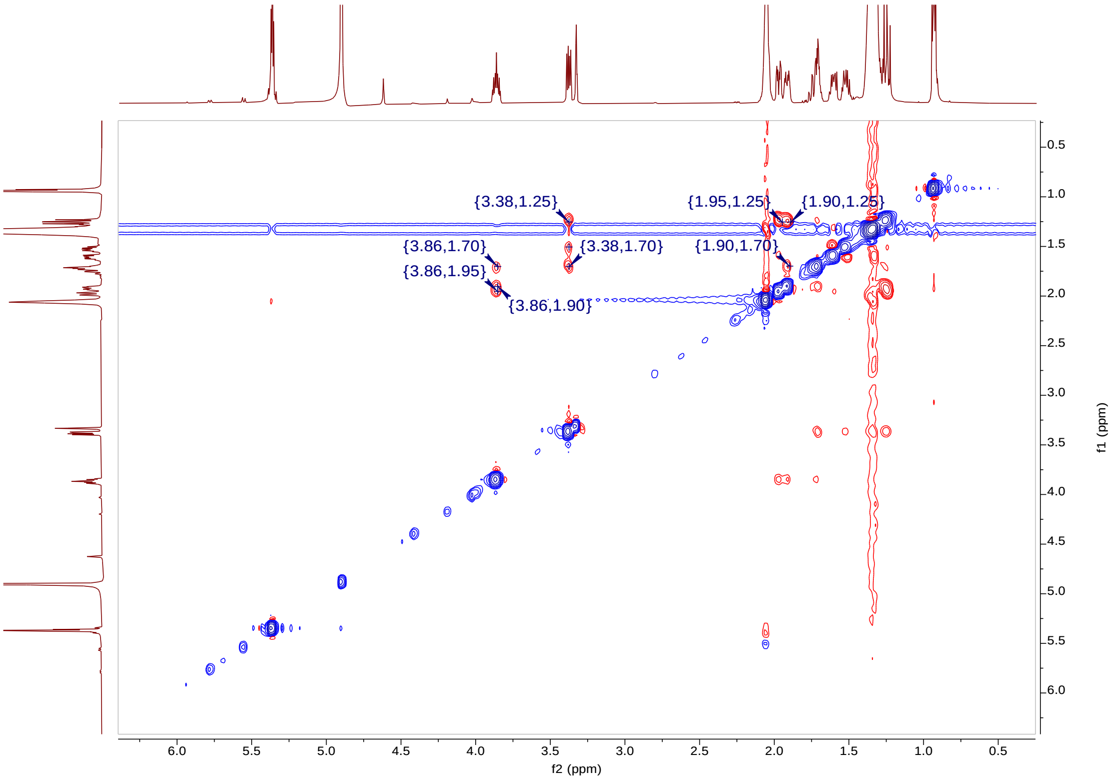

Figure S1.7. HRESIMS spectrum of compound **1**.

Qualitative Analysis Report

**Data Filename**	PQ-6521-.d	**Sample Name**	PQ-6521
**Sample Type**	Sample	**Position**	P1-F6
**Instrument Name**	Instrument 1	**User Name**	
**Acq Method**	s-.m	**Acquired Time**	11/20/2023 11:50:40 AM
**IRM Calibration Status**	Success	**DA Method**	PCDL.m
**Comment**			

Sample Group **Info.**

Acquisition SW Version 6200 series TOF/6500 series Q-TOF B.05.01 (B5125.2)

User Spectra

Fragmentor Voltage	Collision Energy	Ionization Mode
135	0	ESI

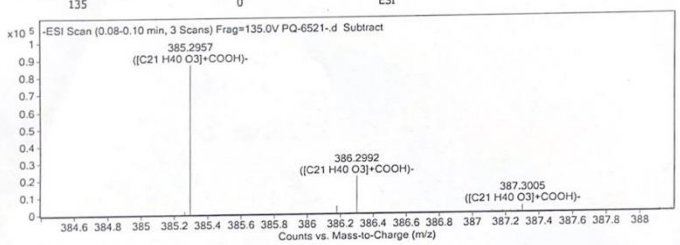

Peak List

m/z	z	Abund	Formula	Ion
61.9886	1	46064.79		
112.9854	1	30170.99		
301.2019	1	70130.85		
375.2712	1	25561.15		
385.2957	1	87130	C21 H40 O3	(M+COOH)-
386.2992	1	21193.77	C21 H40 O3	(M+COOH)-
401.2903	1	20258.72		
416.2647	1	23206.97		
418.2808	1	20902.26		
467.2627	1	18458.09		

Formula Calculator Element Limits

Element	Min	Max
C	3	60
H	0	150
O	0	30

Formula Calculator Results

Formula	CalculatedMass	CalculatedMz	Mz	Diff. (mDa)	Diff. (ppm)	DBE
C21 H40 O3	340.2978	385.2959	385.2957	0.20	0.52	2.0000

--- End Of Report ---

Agilent Technologies Page 1 of 1 Printed at: 11:51 AM on: 11/20/2023

Figure S1.8. UV spectrum of compound **1** in MeOH.

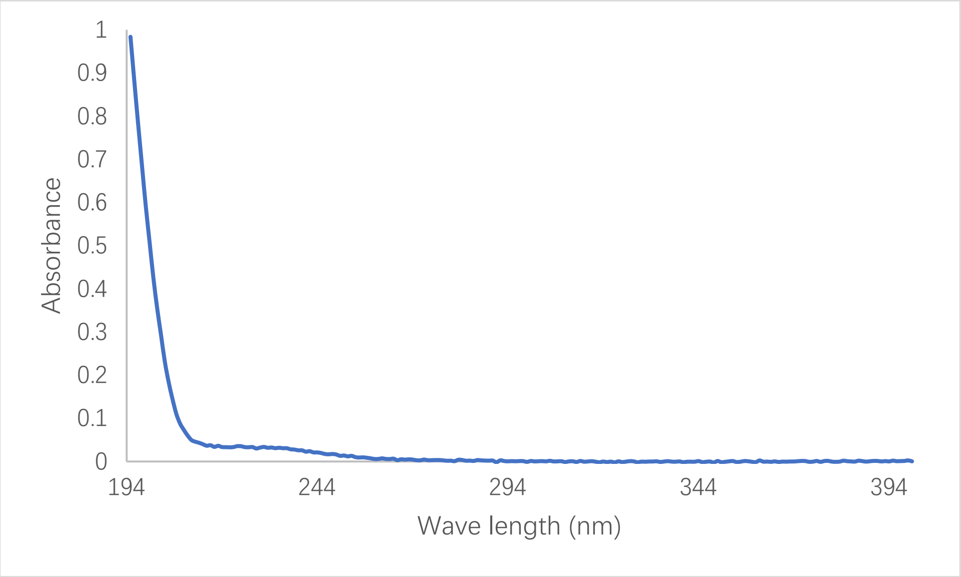

Figure S1.9. IR spectrum of compound **1** in KBr disk.

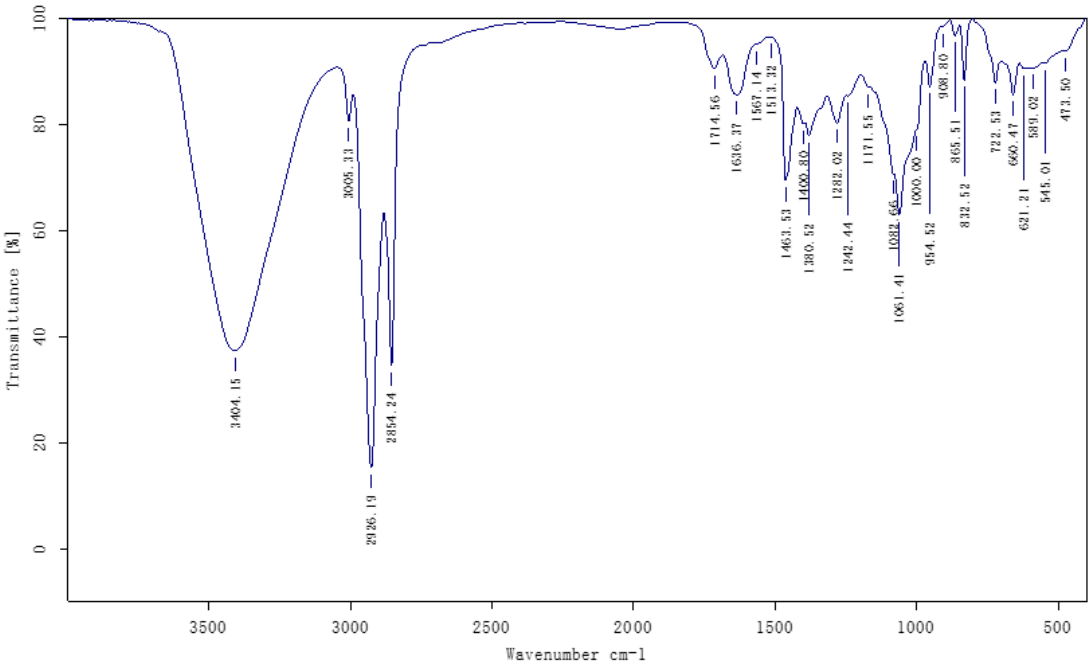

Figure S2.1. ^{1}H NMR spectrum of compound **2** in CD_3OD.

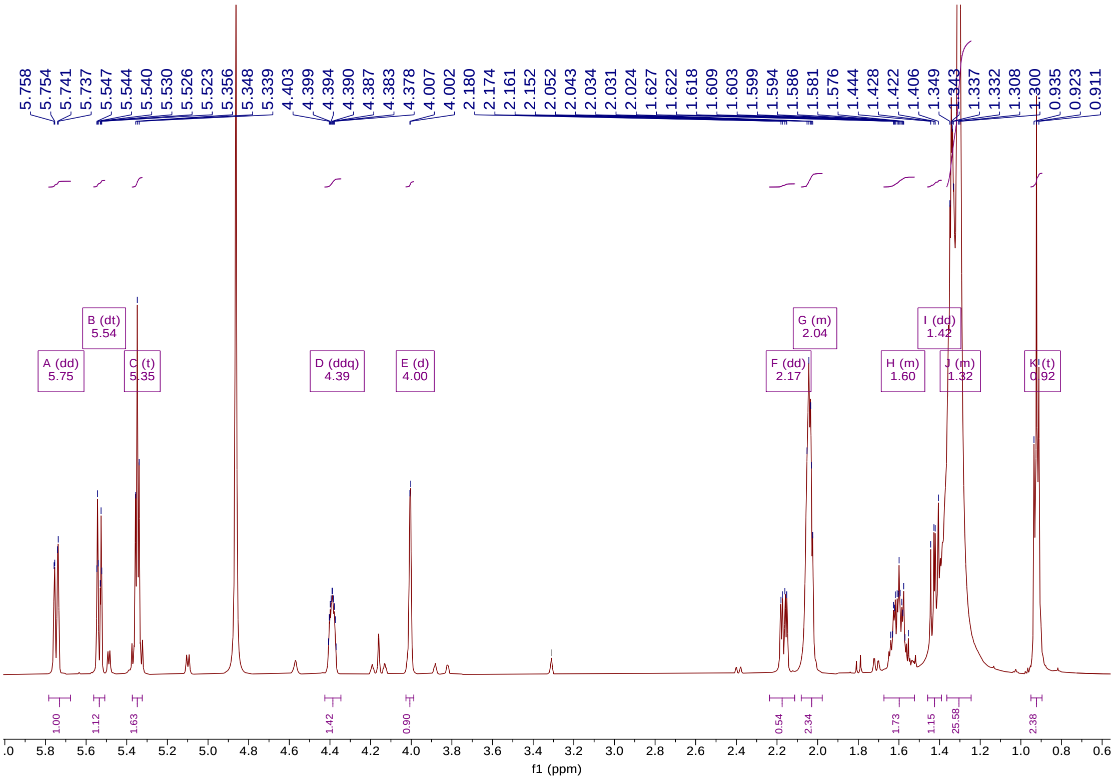

Figure S2.2. ^{13}C NMR and DEPT spectrum of compound **2** in CD_3OD.

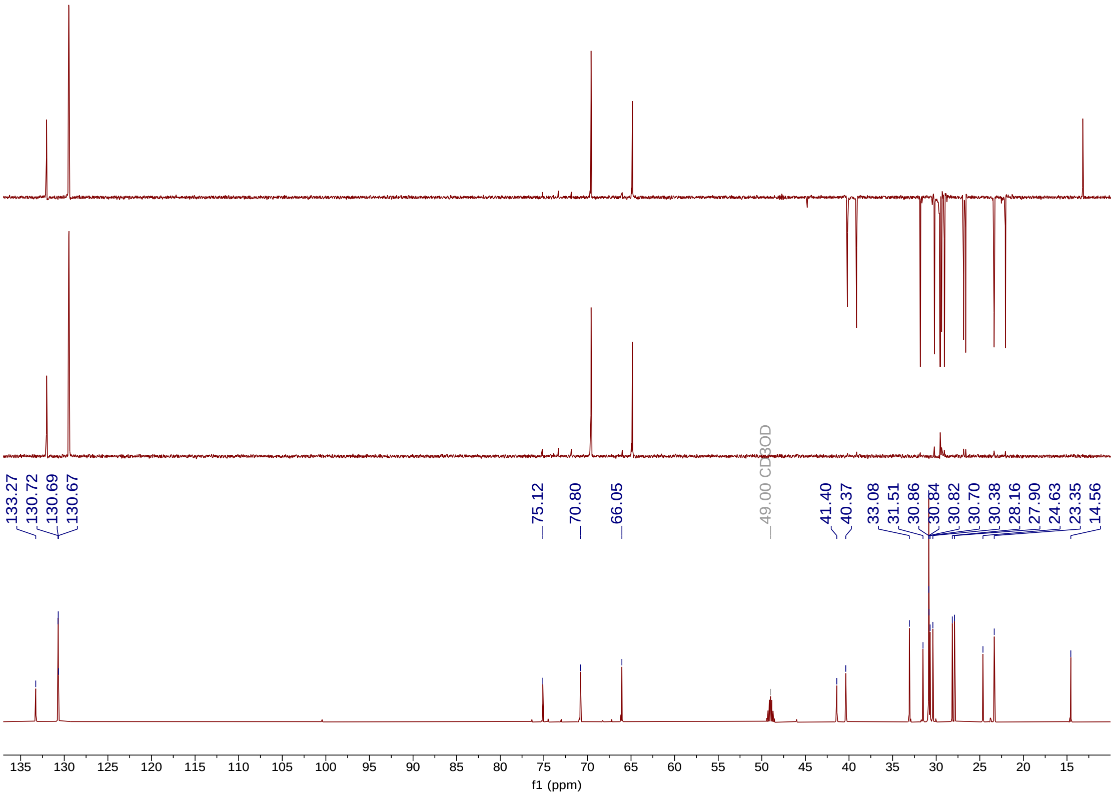

Figure S2.3. ^{1}H-^{1}H COSY spectrum of compound **2** in CD_3OD.

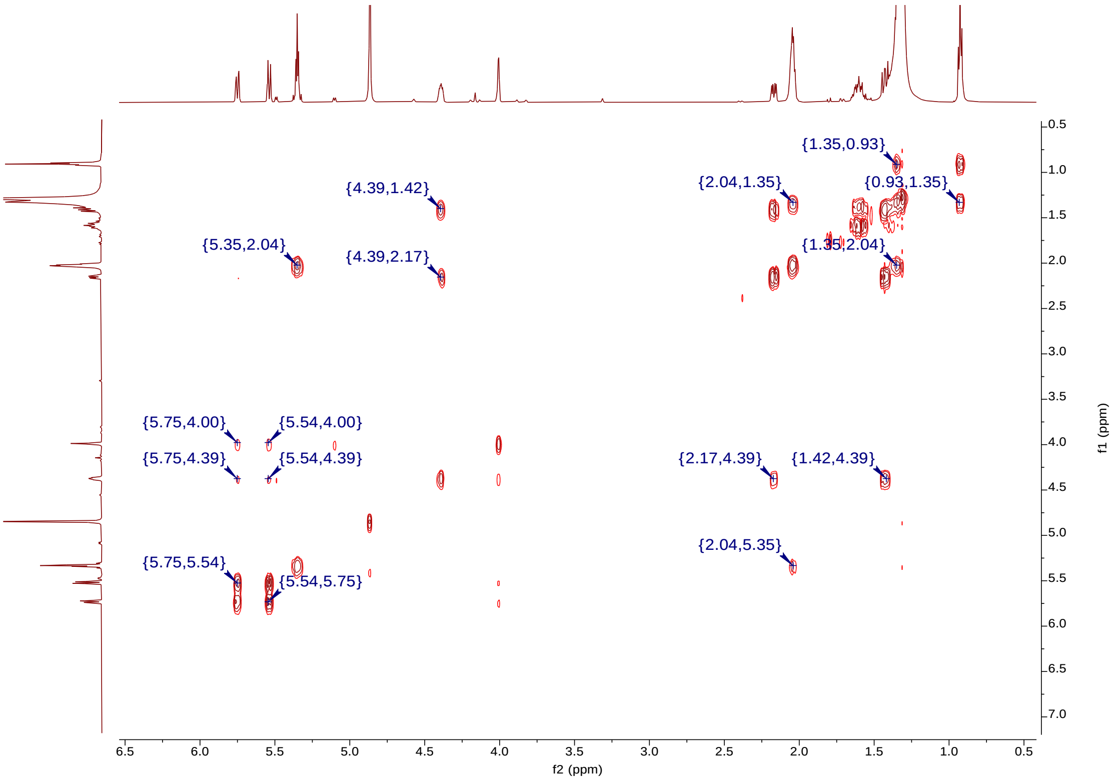

Figure S2.4. HSQC spectrum of compound **2** in CD_3OD.

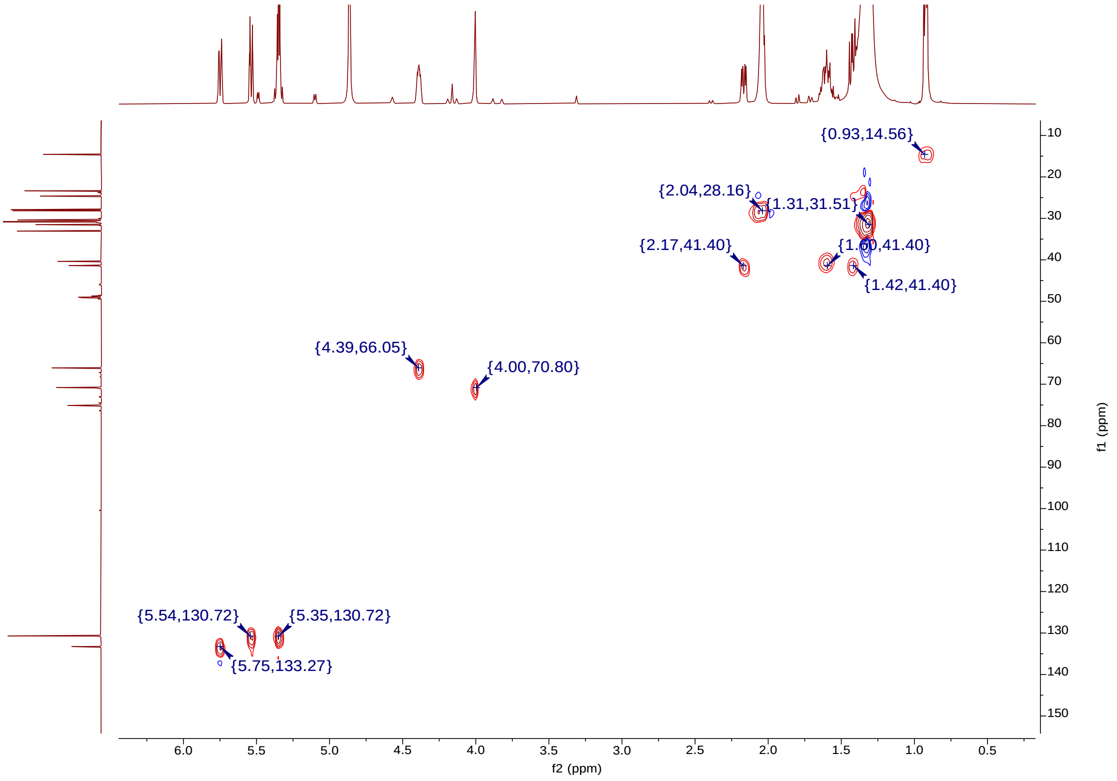

Figure S2.5. HMBC spectrum of compound **2** in CD_3OD.

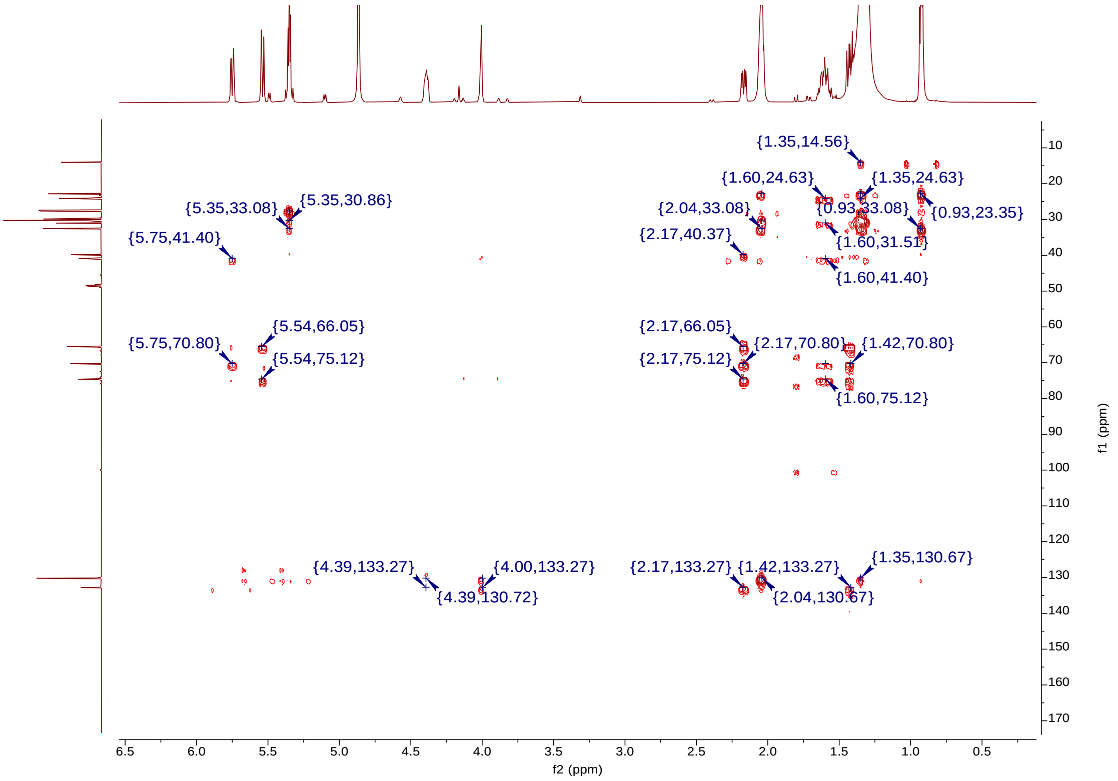

Figure S2.6. ROESY spectrum of compound **2** in CD_3OD.

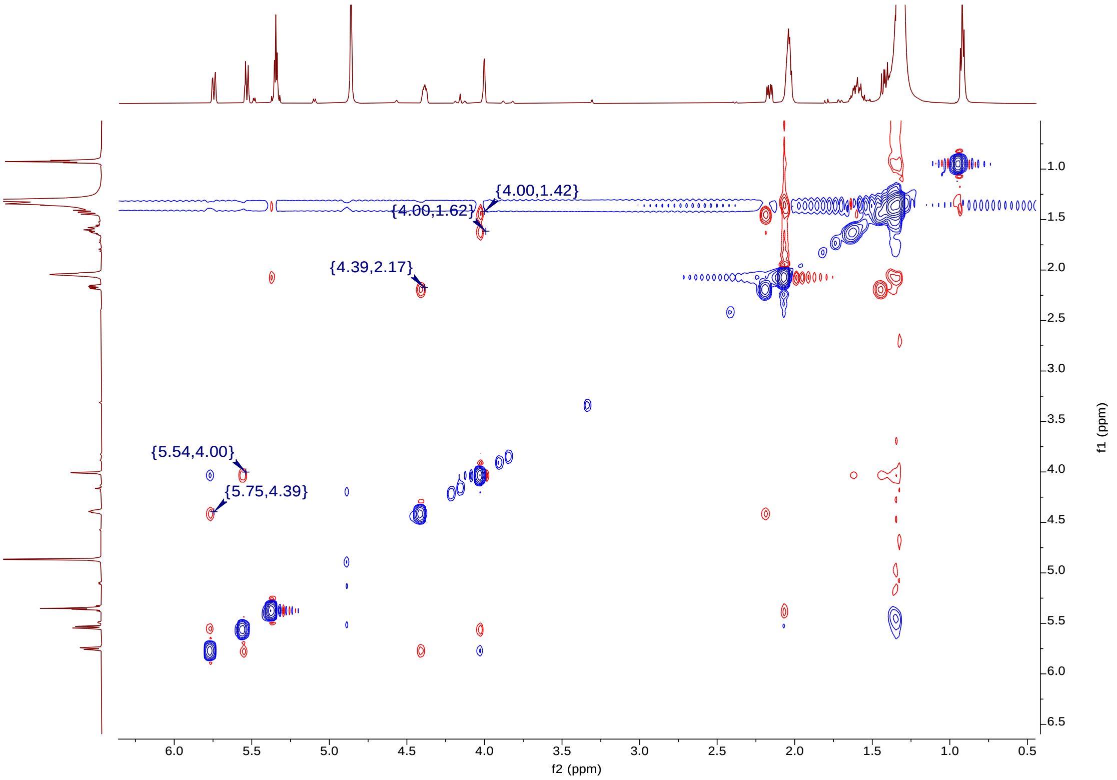

Figure S2.7. HRESIMS spectrum of compound **2**.

Qualitative Analysis Report

**Data Filename**	PQ-6517.d	**Sample Name**	PQ-6517
**Sample Type**	Sample	**Position**	P1-F5
**Instrument Name**	Instrument 1	**User Name**	
**Acq Method**	s.m	**Acquired Time**	11/20/2023 11:34:19 AM
**IRM Calibration Status**	Success	**DA Method**	PCDL.m
**Comment**			

Sample Group **Info.**
Acquisition SW Version 6200 series TOF/6500 series Q-TOF B.05.01 (B5125.2)

User Spectra

Fragmentor Voltage	Collision Energy	Ionization Mode
135	0	ESI

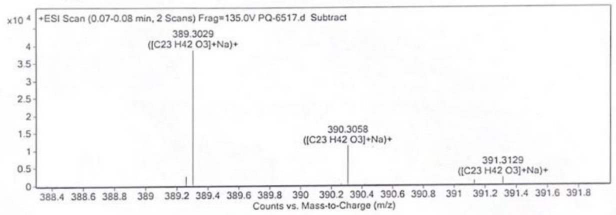

Peak List

m/z	z	Abund	Formula	Ion
388.2544	1	11997.78		
389.3029	1	38839.07	C23 H42 O3	(M+Na)+
390.3058	1	11249.73	C23 H42 O3	(M+Na)+
421.2928	1	12876.43		
432.2804	1	13795.59		
437.236	1	9450.2		
476.3072	1	14491.48		
481.262	1	8608.04		
520.3327	1	11400.15		
564.3598	1	8507.87		

Formula Calculator Element Limits

Element	Min	Max
C	3	150
H	0	300
O	0	30

Formula Calculator Results

Formula	CalculatedMass	CalculatedMz	Mz	Diff. (mDa)	Diff. (ppm)	DBE
C23 H42 O3	366.3134	389.3026	389.3029	-0.30	-0.77	3.0000

--- End Of Report ---

Agilent Technologies Page 1 of 1 Printed at: 11:44 AM on: 11/20/2023

Figure S2.8. UV spectrum of compound **2** in MeOH.

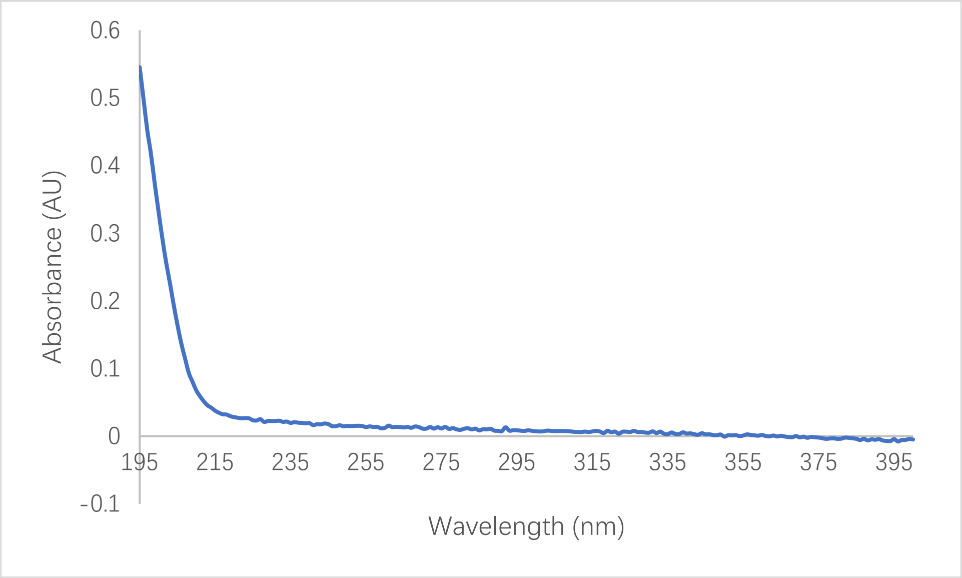

Figure S2.9. IR spectrum of compound **2** in KBr disk.

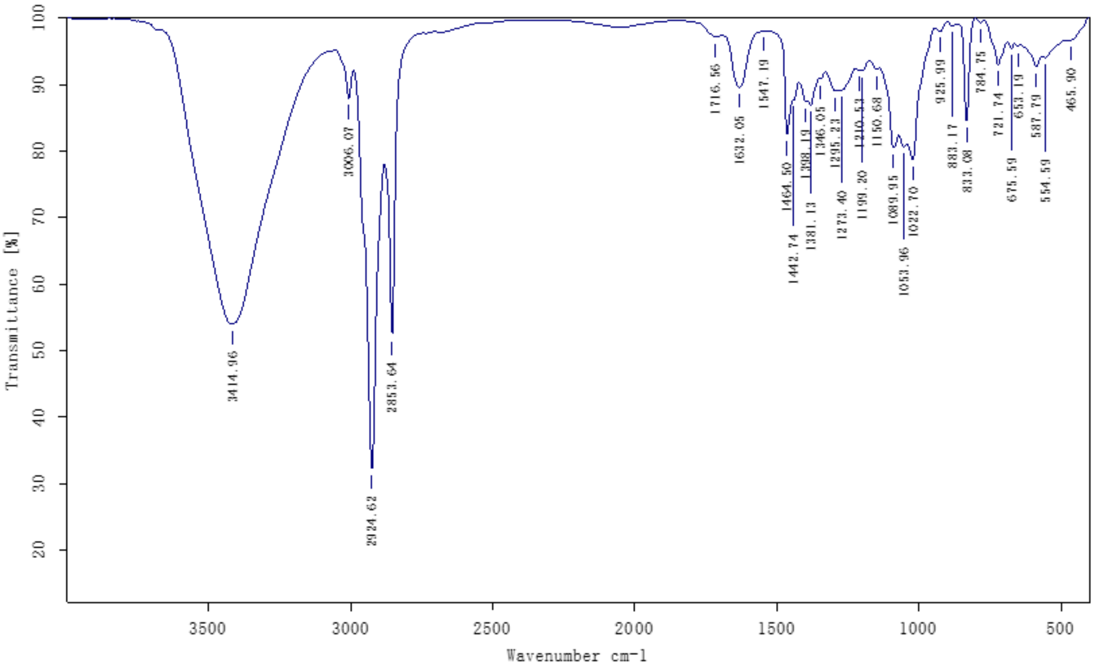

Figure S3.1. ^{1}H NMR spectrum of compound **3** in CD_3OD.

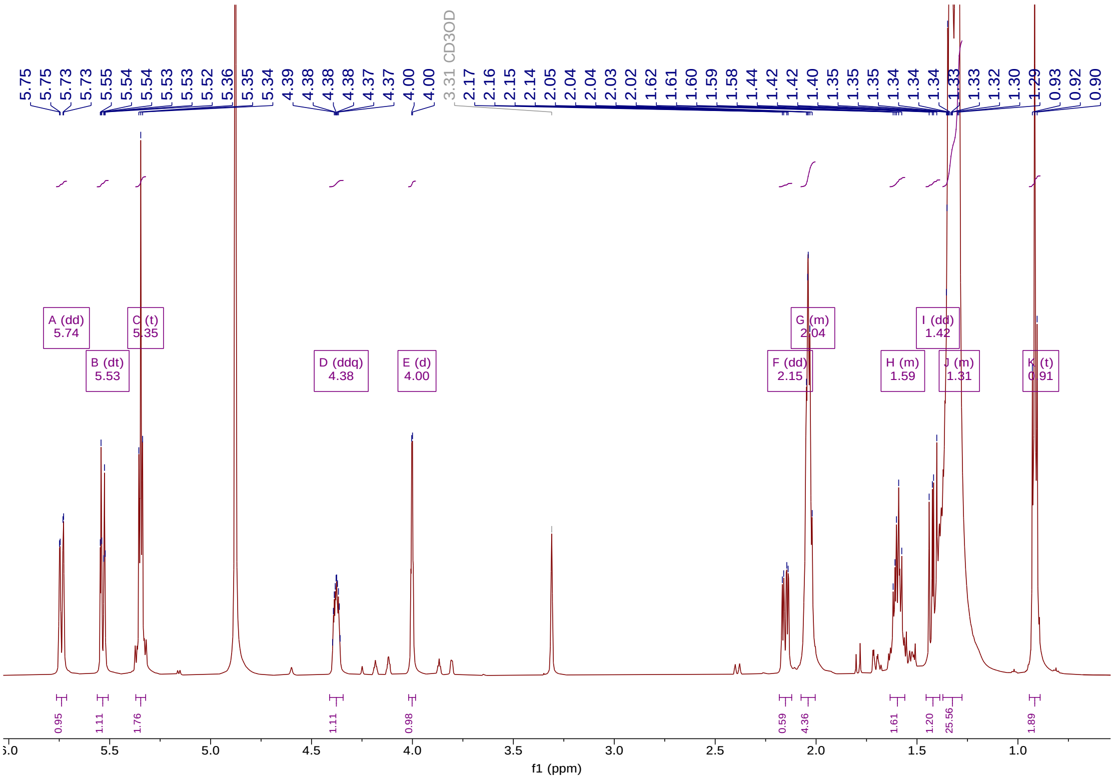

Figure S3.2. ^{13}C NMR and DEPT spectrum of compound **3** in CD_3COCD_3.

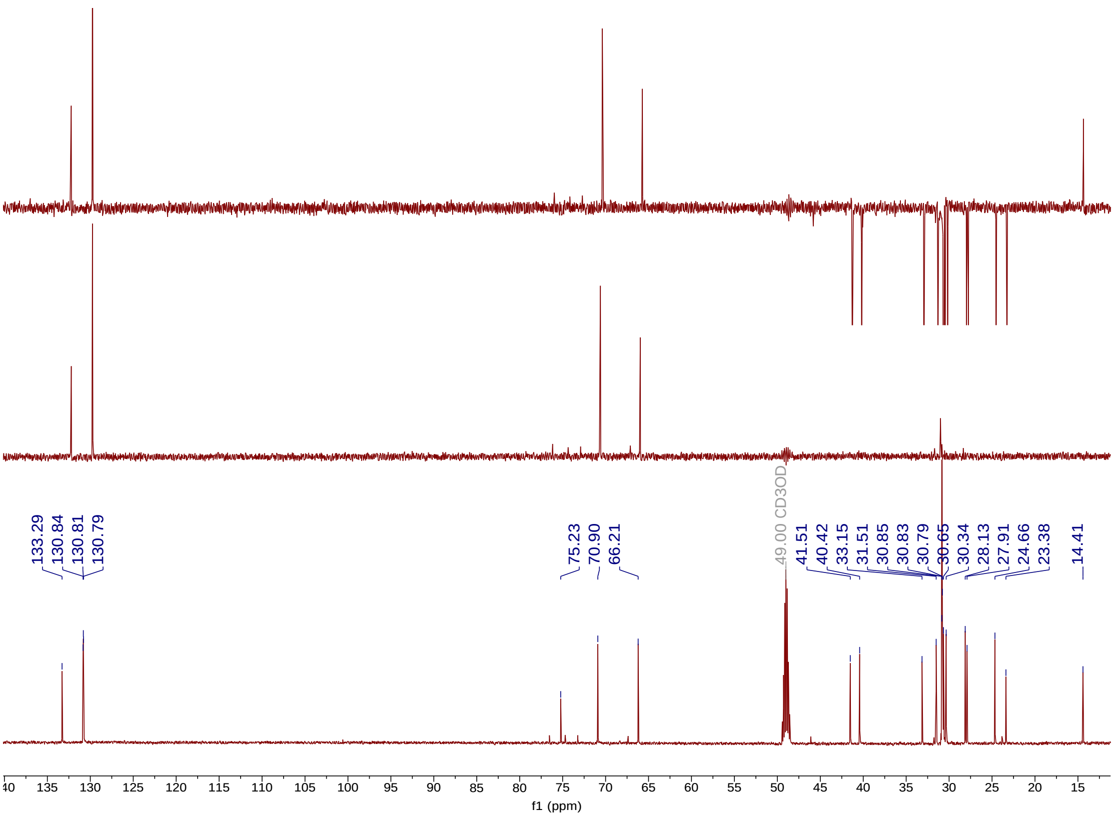

Figure S3.3. ^{1}H-^{1}H COSY spectrum of compound **3** in CD_3OD.

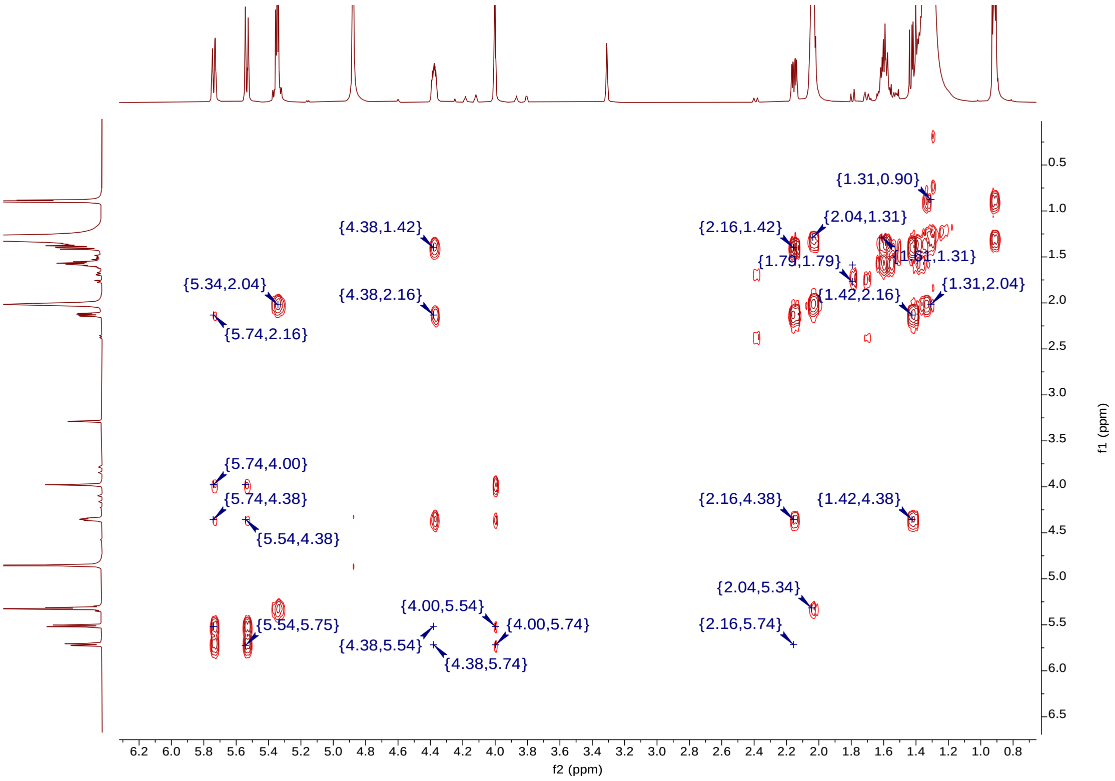

Figure S3.4. HSQC spectrum of compound **3** in CD_3OD.

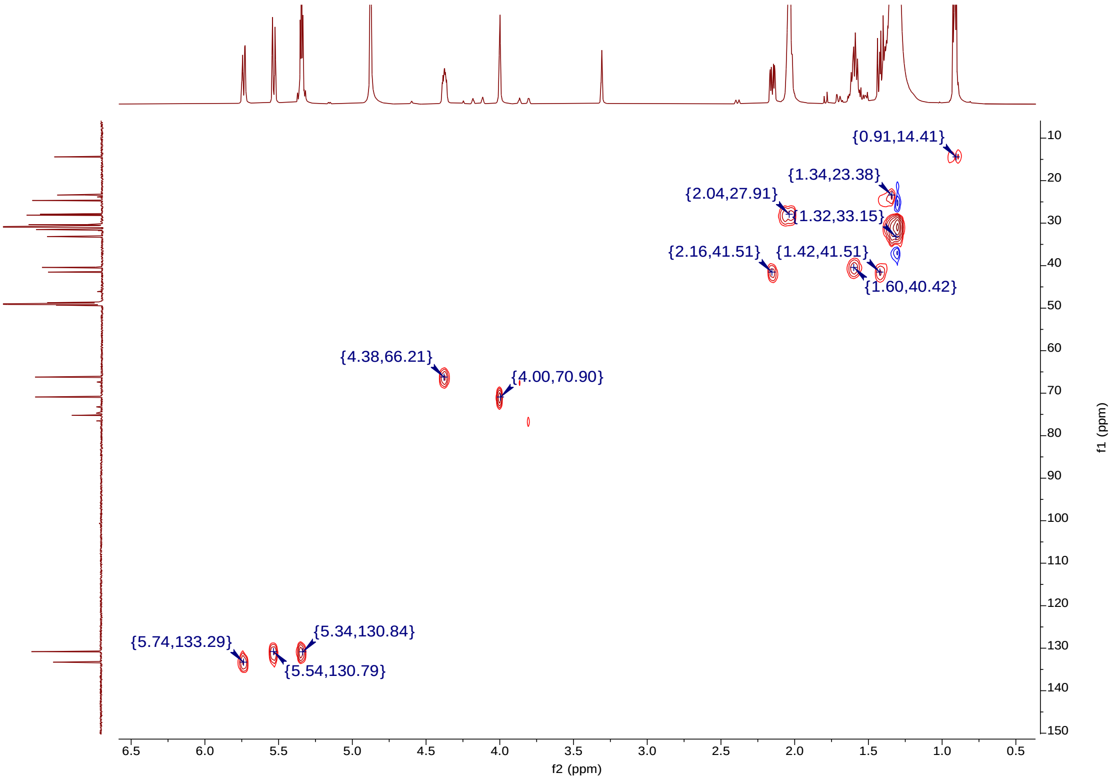

Figure S3.5. HMBC spectrum of compound **3** in CD_3OD.

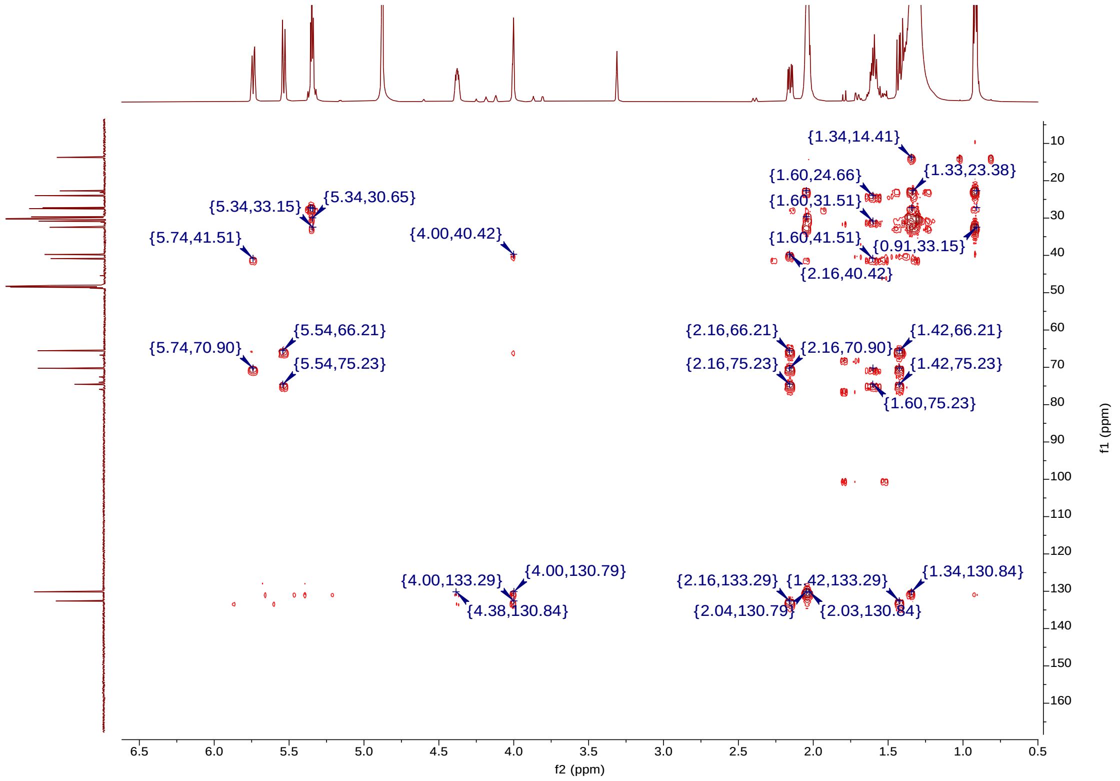

Figure S3.6. ROESY spectrum of compound **3** in CD_3OD.

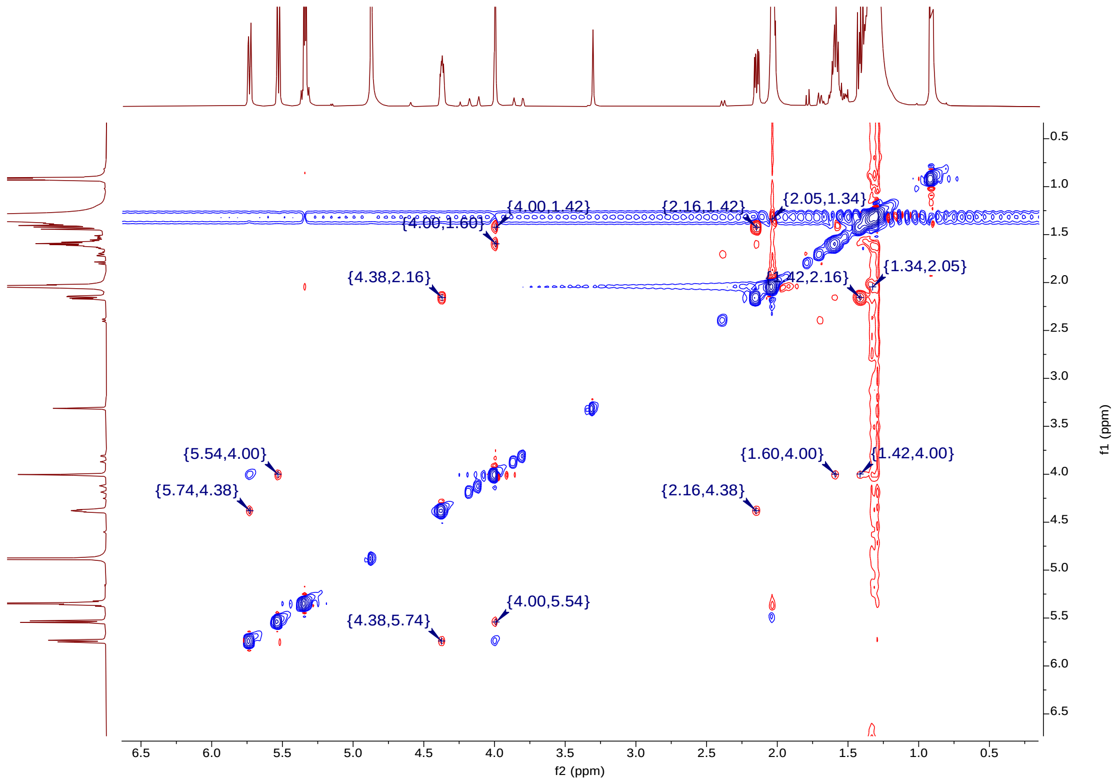

Figure S3.7. HRESIMS Spectrum of compound **3**.

Qualitative Analysis Report

**Data Filename**	PQ-6516.d	**Sample Name**	PQ-6516
**Sample Type**	Sample	**Position**	P1-F4
**Instrument Name**	Instrument 1	**User Name**	
**Acq Method**	s.m	**Acquired Time**	11/20/2023 11:33:10 AM
**IRM Calibration Status**	Success	**DA Method**	PCDL.m
**Comment**			

Sample Group **Info.**
Acquisition SW Version 6200 series TOF/6500 series Q-TOF B.05.01 (B5125.2)

User Spectra

Fragmentor Voltage	Collision Energy	Ionization Mode
135	0	ESI

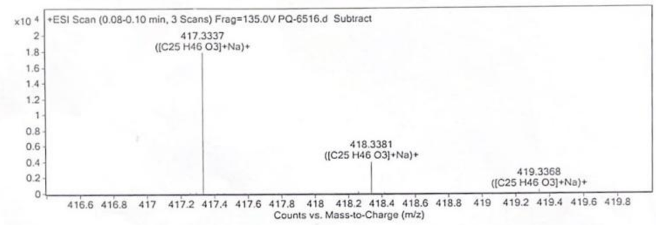

Peak List

m/z	z	Abund
344.2282	1	32498.18
349.1834	1	35453.77
388.2547	1	49604.6
393.2095	1	41085.96
432.2806	1	52759.75
437.2358	1	46332.46
476.3069	1	52572.41
481.2622	1	37165.83
520.3333	1	37446.12
525.2887	1	27264.02

Formula Calculator Element Limits

Element	Min	Max
C	3	150
H	0	300
O	0	30

Formula Calculator Results

Formula	CalculatedMass	CalculatedMz	Mz	Diff. (mDa)	Diff. (ppm)	DBE
C25 H46 O3	394.3447	417.3339	417.3337	0.20	0.48	3.0000

--- End Of Report ---

Agilent Technologies Page 1 of 1 Printed at: 11:43 AM on: 11/20/2023

Figure S3.8. UV Spectrum of compound **3** in MeOH.

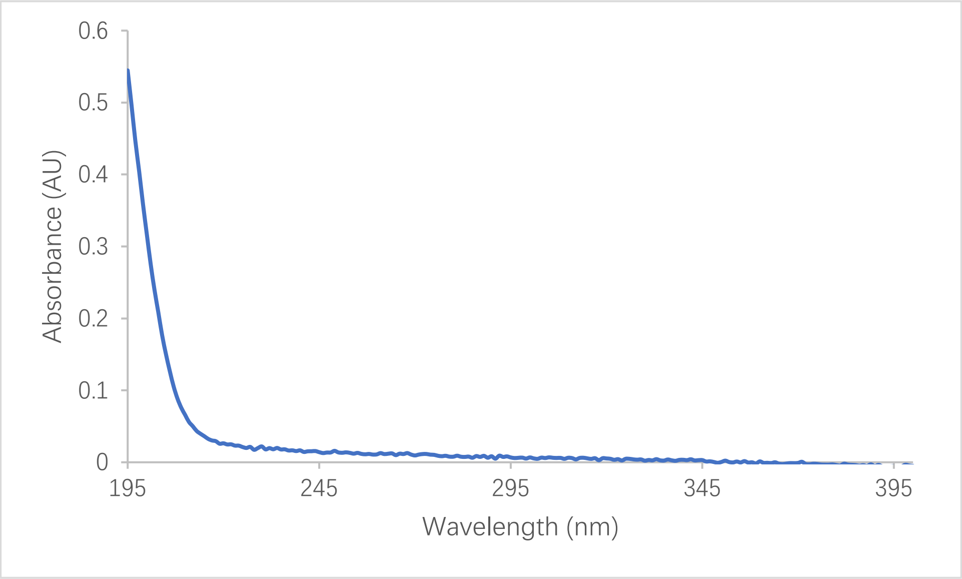

Figure S3.9. IR Spectrum of compound **3** in KBr disk.

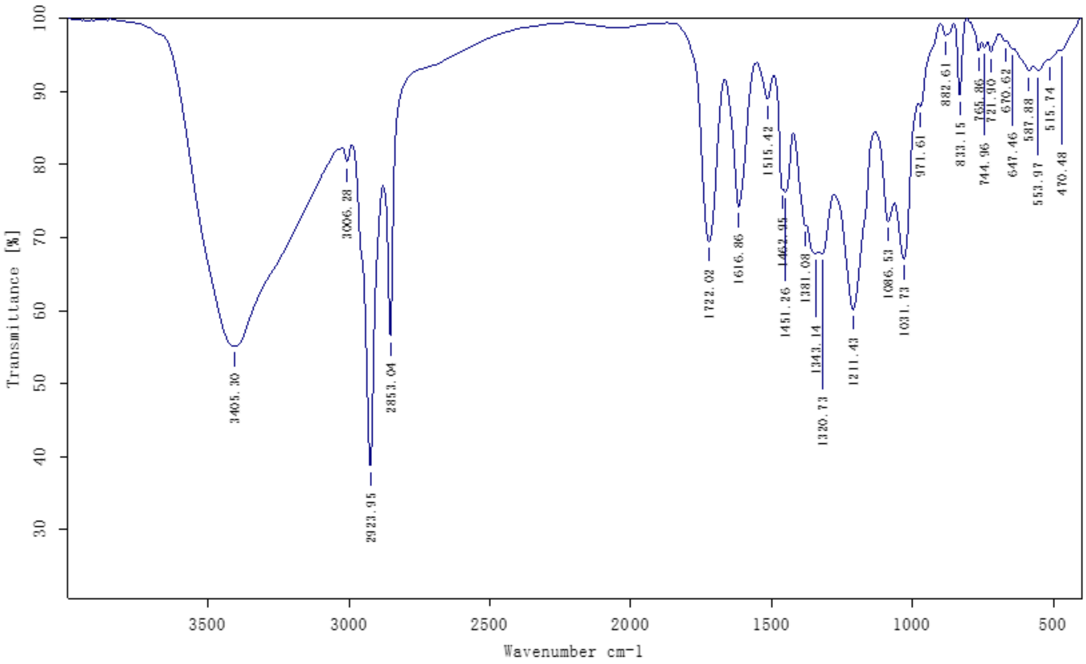

Figure S4.1. ^{1}H NMR spectrum of compound **4** in CD_3OD.

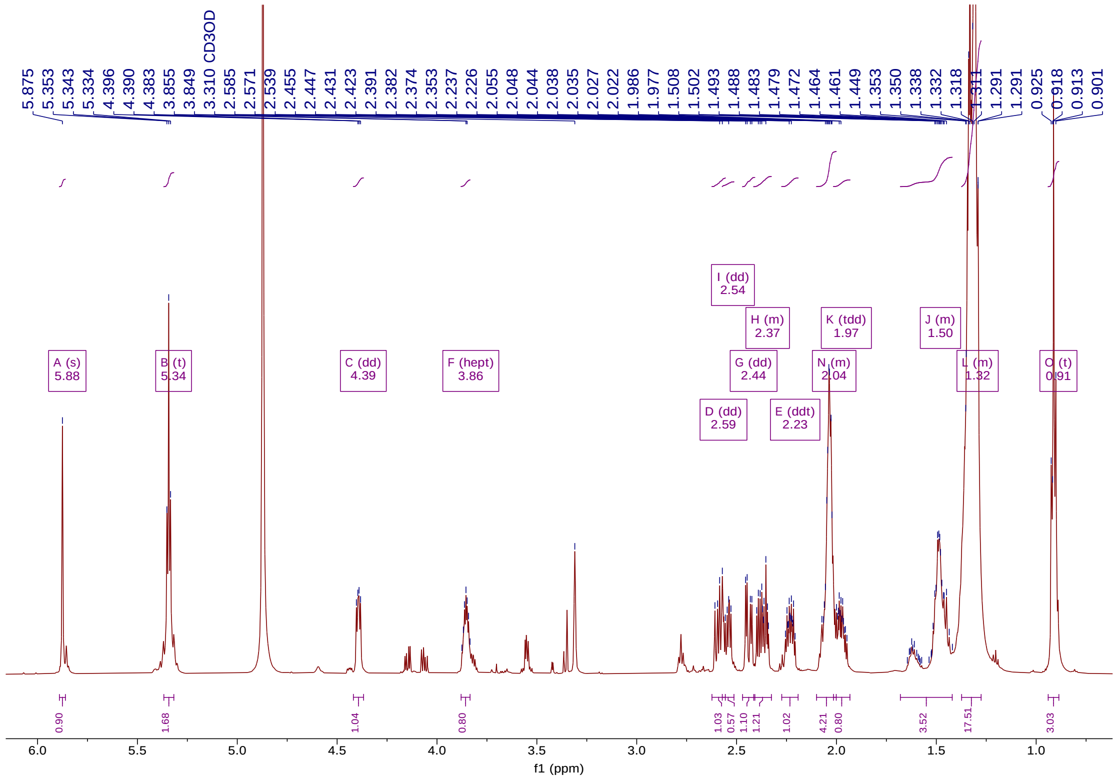

Figure S4.2. ^{13}C NMR and DEPT spectrum of compound **4** in CD_3OD.

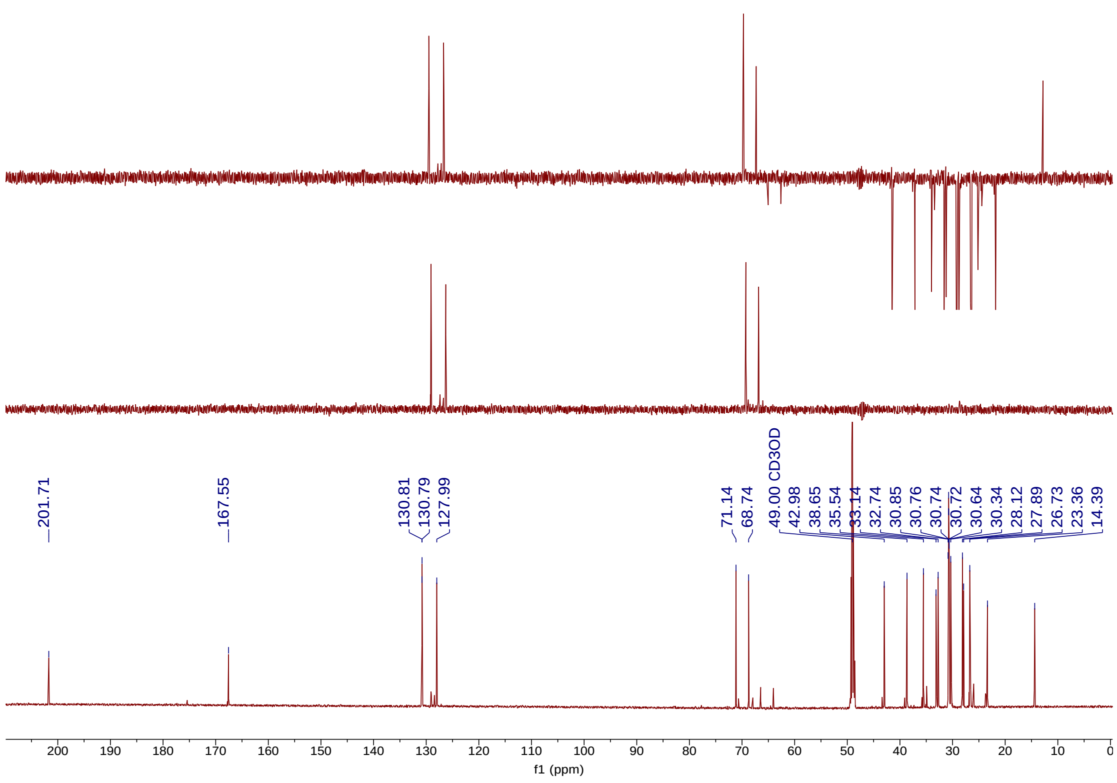

Figure S4.3. ^{1}H-^{1}H COSY spectrum of compound **4** in CD_3OD.

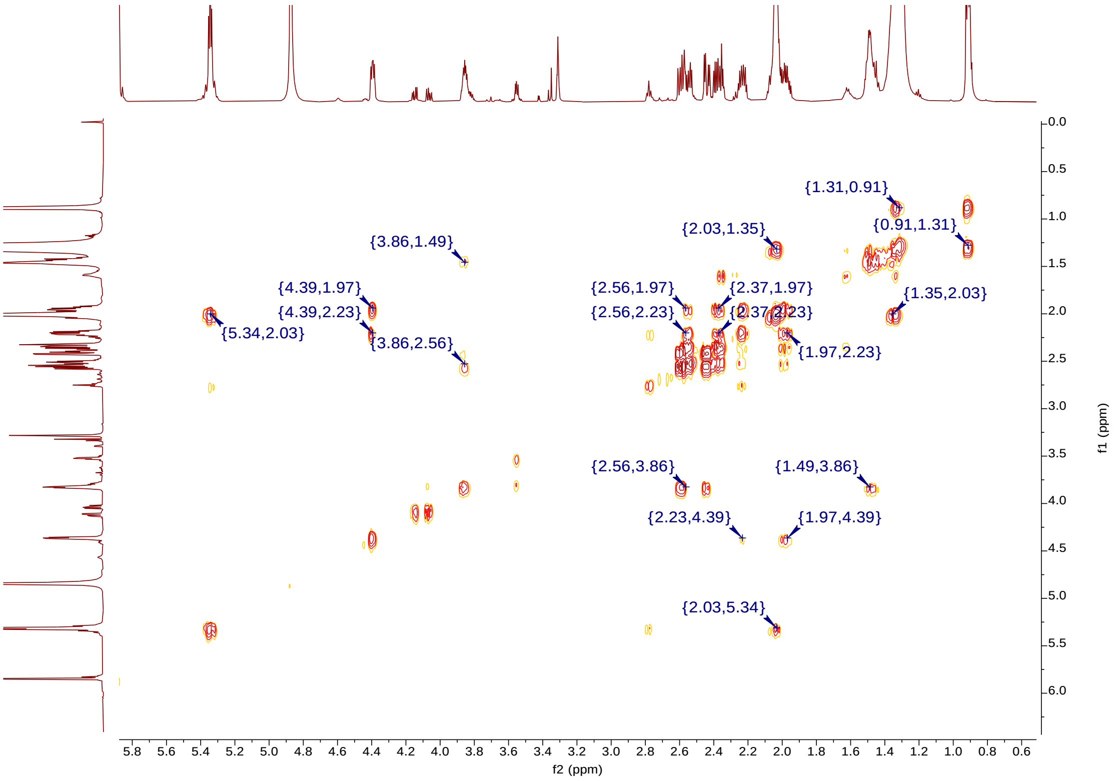

Figure S4.4. HSQC spectrum of compound **4** in CD_3OD.

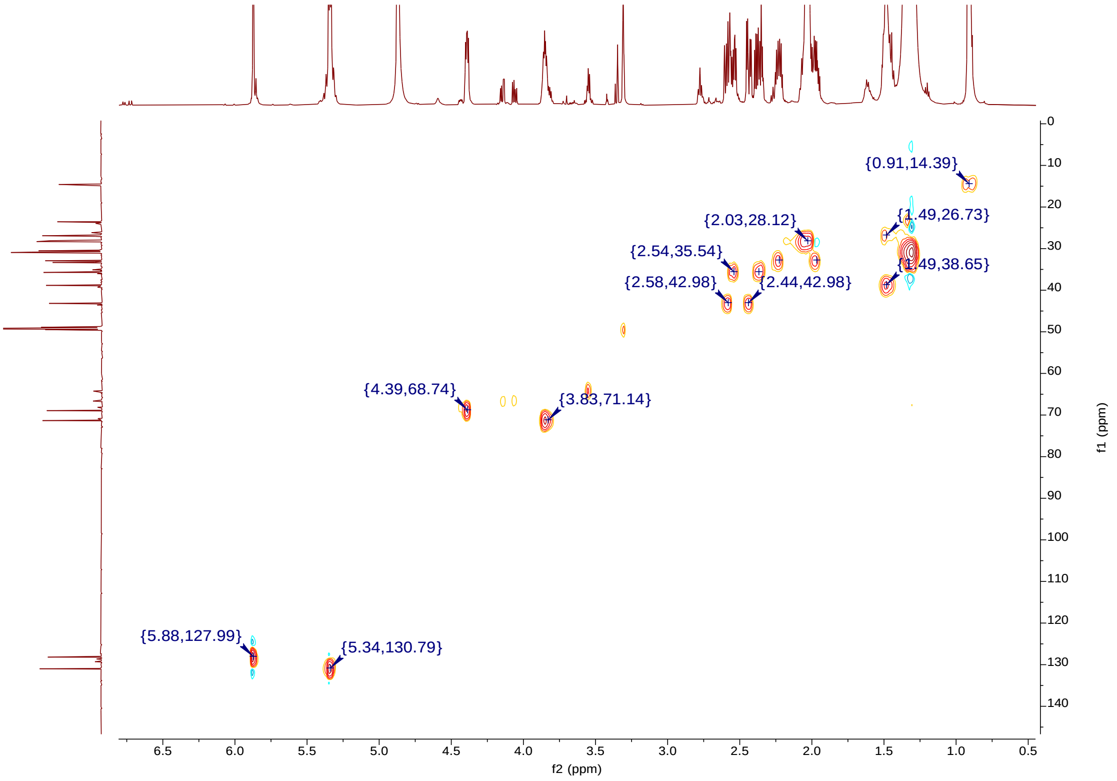

Figure S4.5. HMBC spectrum of compound **4** in CD_3OD.

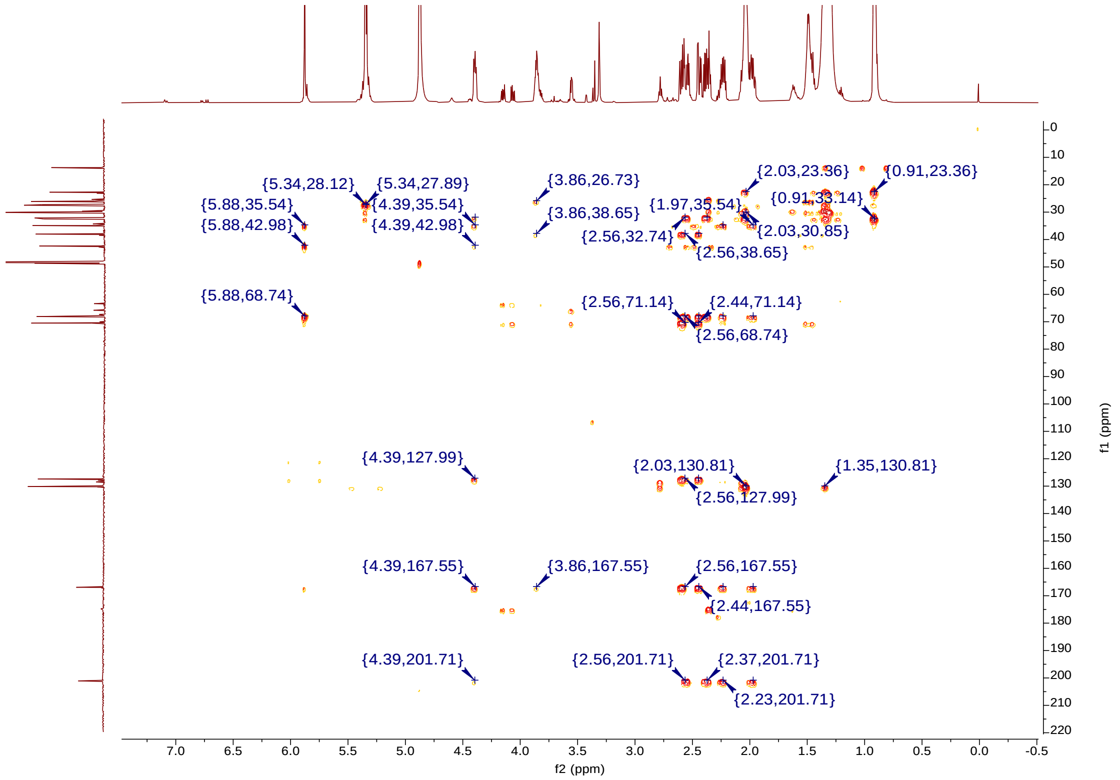

Figure S4.6. ROESY spectrum of compound **4** in CD_3OD.

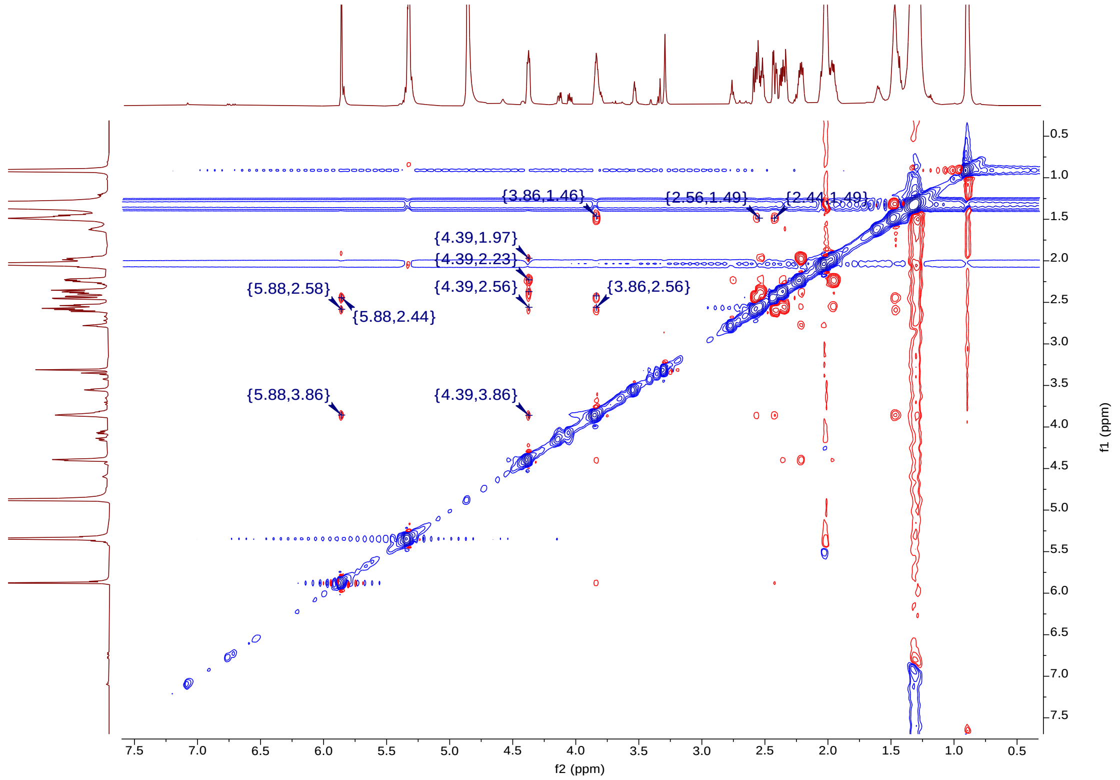

Figure S4.7. HRESIMS spectrum of compound **4**.

Qualitative Analysis Report

**Data Filename**	PQ-6515+.d	**Sample Name**	PQ-6515
**Sample Type**	Sample	**Position**	P1-F3
**Instrument Name**	Instrument 1	**User Name**	
**Acq Method**	s.m	**Acquired Time**	11/20/2023 11:40:51 AM
**IRM Calibration Status**	Success	**DA Method**	PCDL.m
**Comment**			

**Sample Group**		**Info.**
**Acquisition SW Version**	6200 series TOF/6500 series Q-TOF B.05.01 (B5125.2)	

User Spectra

Fragmentor Voltage	Collision Energy	Ionization Mode
135	0	ESI

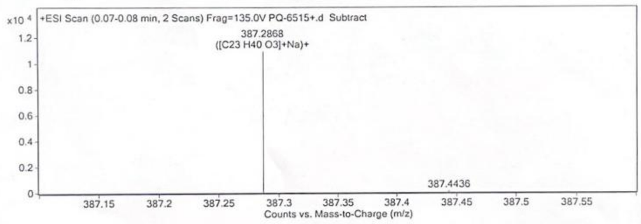

Peak List

m/z	z	Abund	Formula	Ion
305.1569	1	9338.34		
349.1837	1	19438.09		
387.2868	1	10953.29	C23 H40 O3	(M+Na)+
393.2098	1	28739.12		
437.2361	1	30702.42		
481.2623	1	28056.61		
524.3349	1	11363.59		
525.2889	1	19817.04		
569.3149	1	12738.58		
751.5862	1	17422.55		

Formula Calculator Element Limits

Element	Min	Max
C	3	150
H	0	300
O	0	30

Formula Calculator Results

Formula	CalculatedMass	CalculatedMz	Mz	Diff. (mDa)	Diff. (ppm)	DBE
C23 H40 O3	364.2978	387.2870	387.2868	0.20	0.52	4.0000

--- End Of Report ---

Agilent Technologies Page 1 of 1 Printed at: 11:42 AM on: 11/20/2023

Figure S4.8. UV spectrum of compound **4** in MeOH.

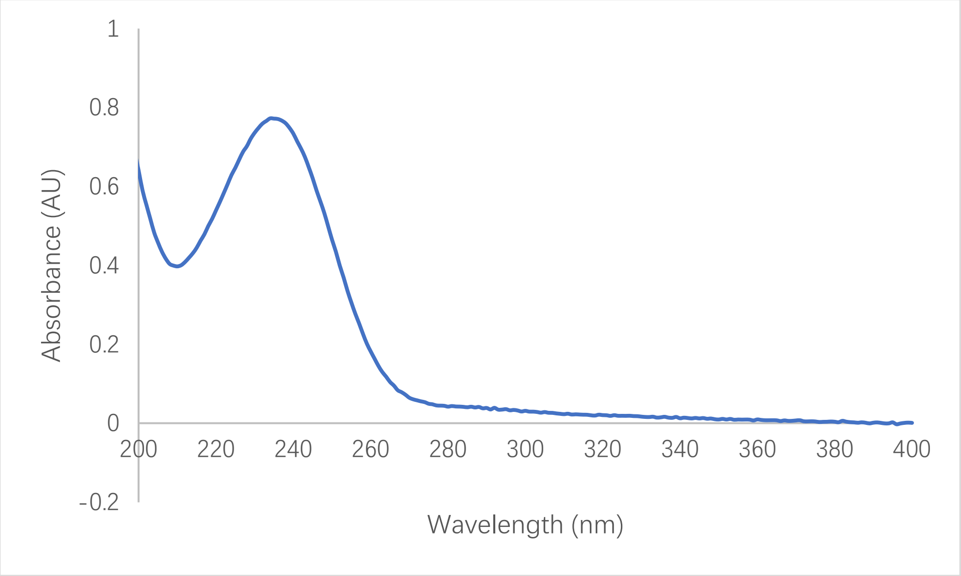

Figure S4.9. IR spectrum of compound **4** in KBr disk.

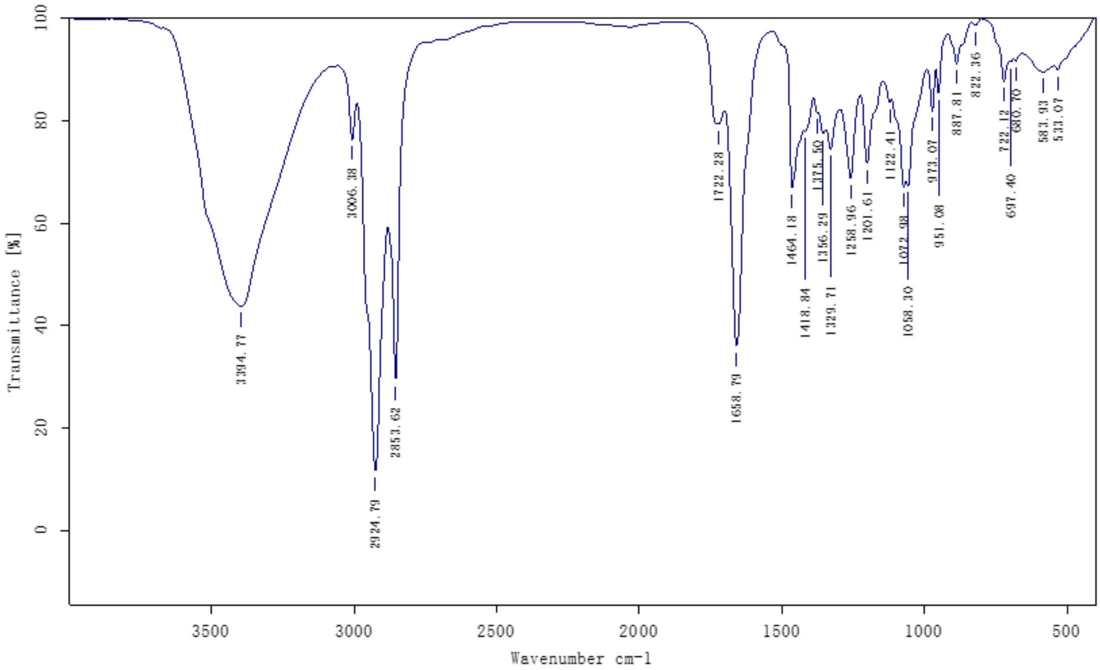

Figure S5.1. ^{1}H NMR spectrum of compound **5** in CD_3OD.

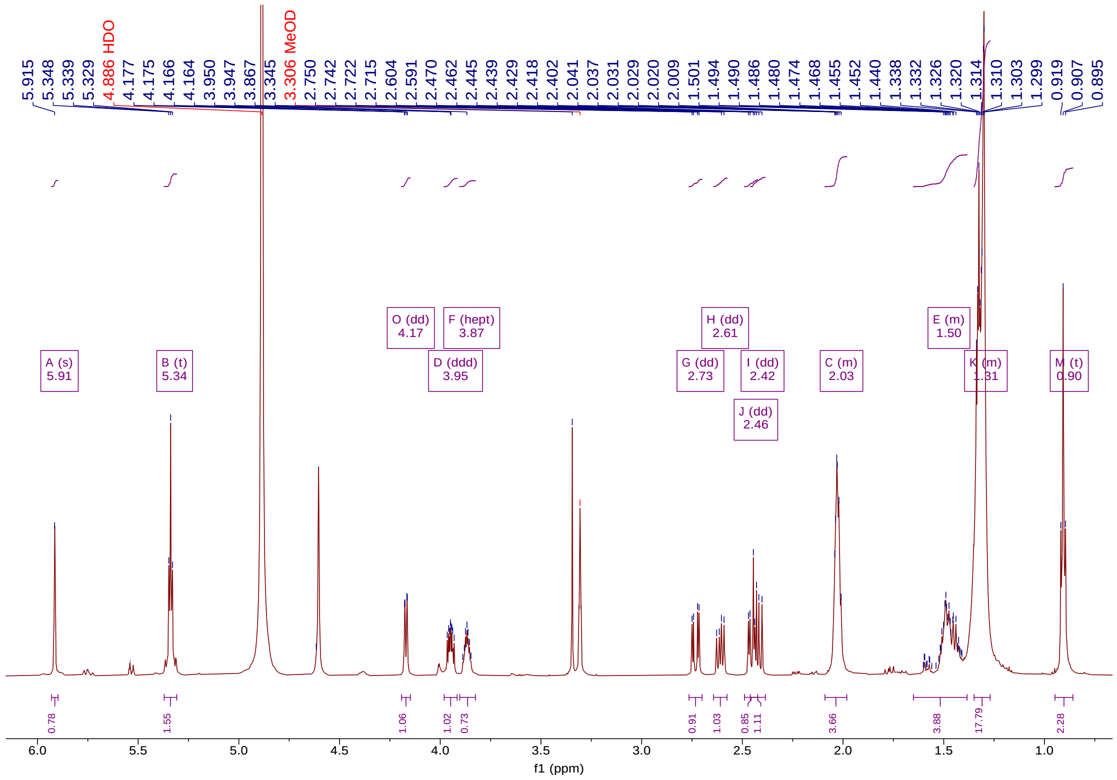

Figure S5.2. ^{13}C NMR and DEPT spectrum of compound **5** in CD_3OD.

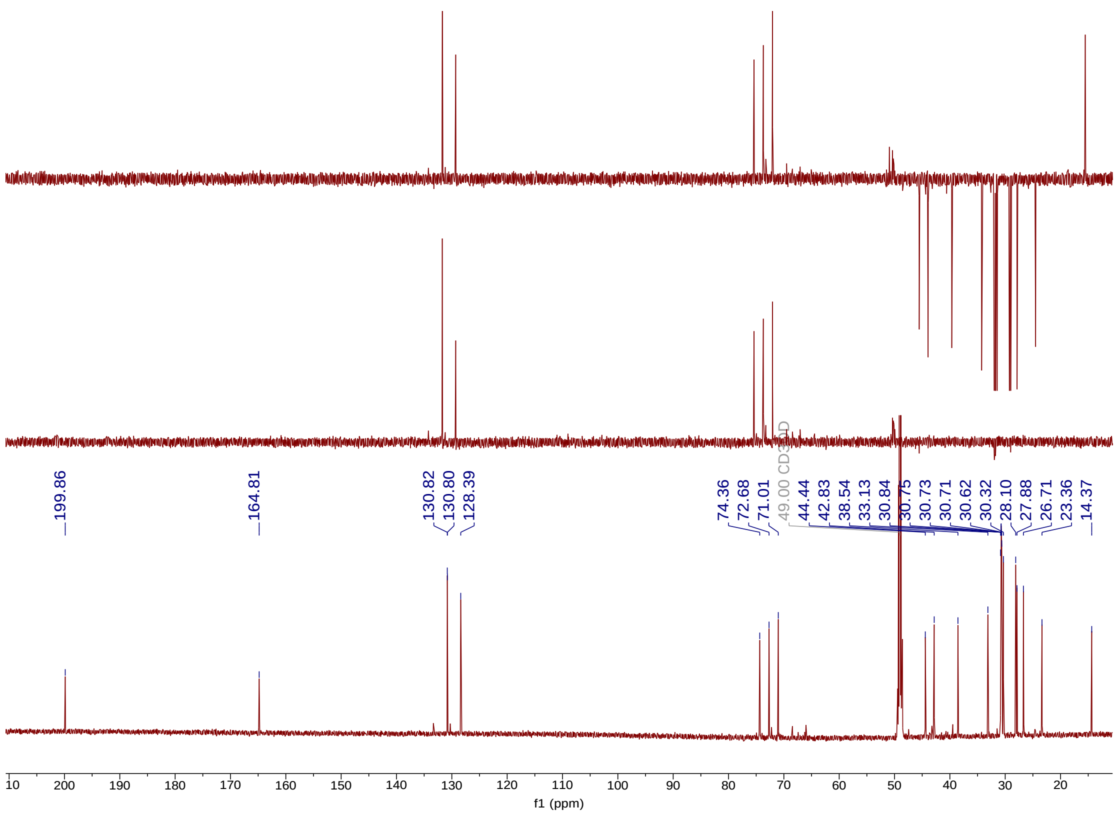

Figure S5.3. ^{1}H-^{1}H COSY spectrum of compound **5** in CD_3OD.

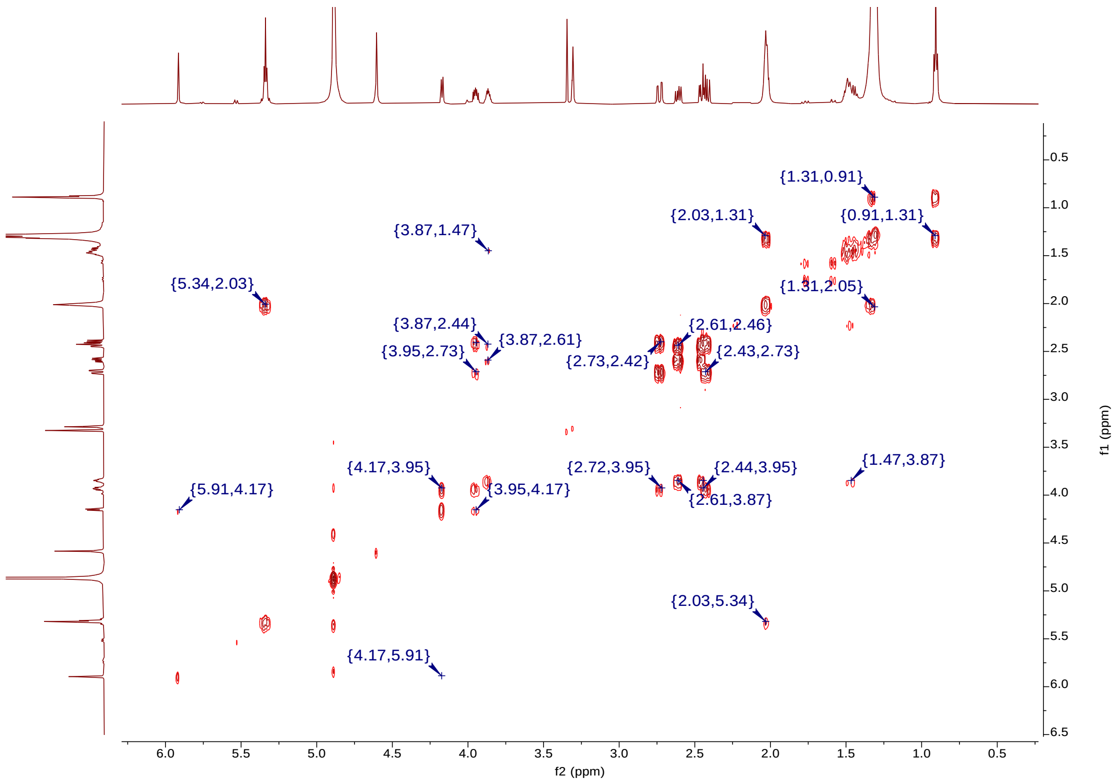

Figure S5.4. HSQC spectrum of compound **5** in CD_3OD.

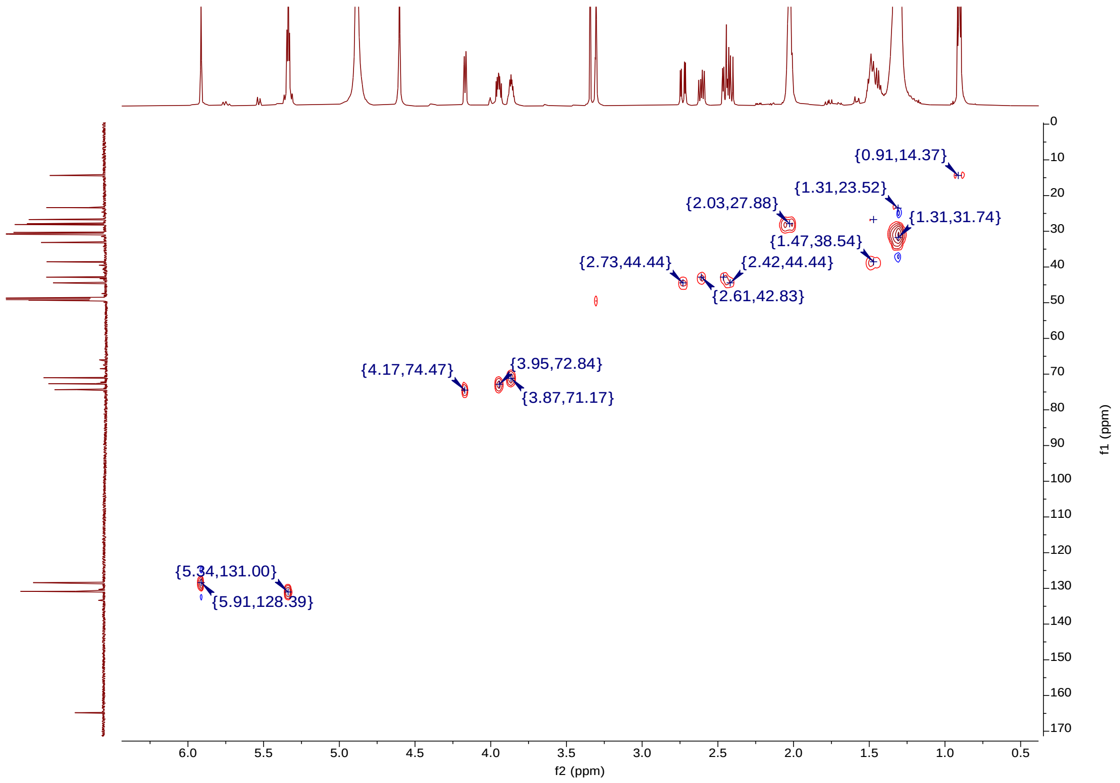

Figure S5.5. HMBC spectrum of compound **5** in CD_3OD.

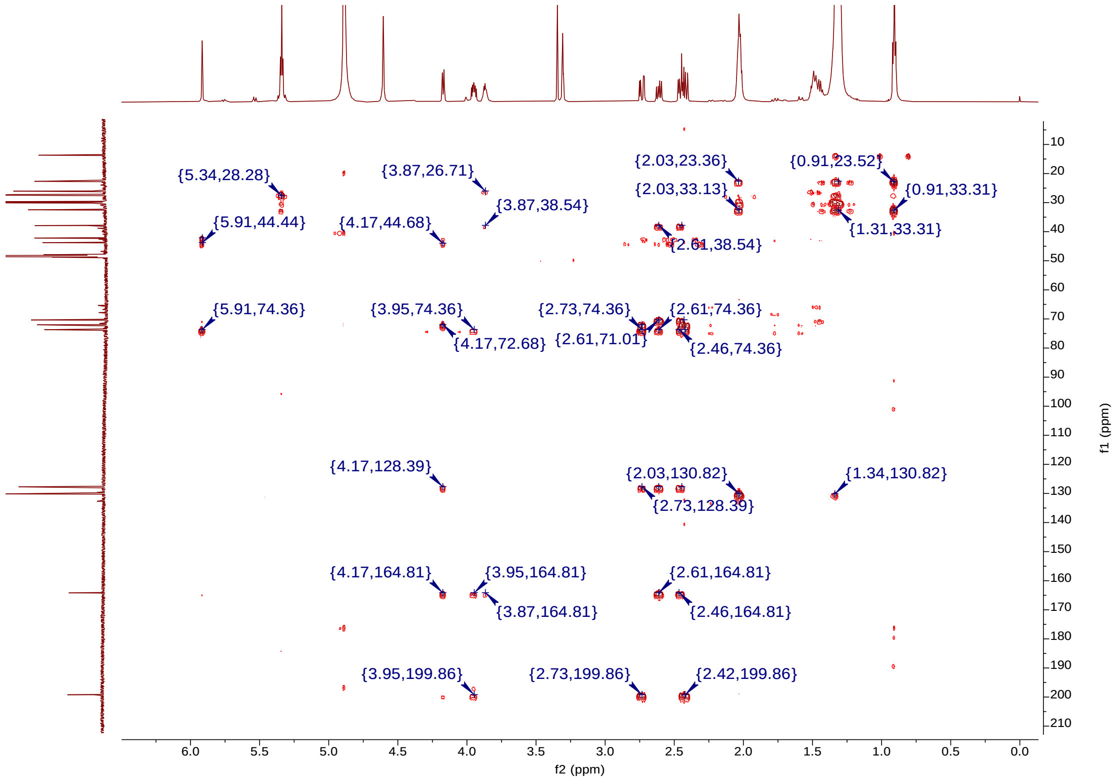

Figure S5.6. ROESY spectrum of compound **5** in CD_3OD.

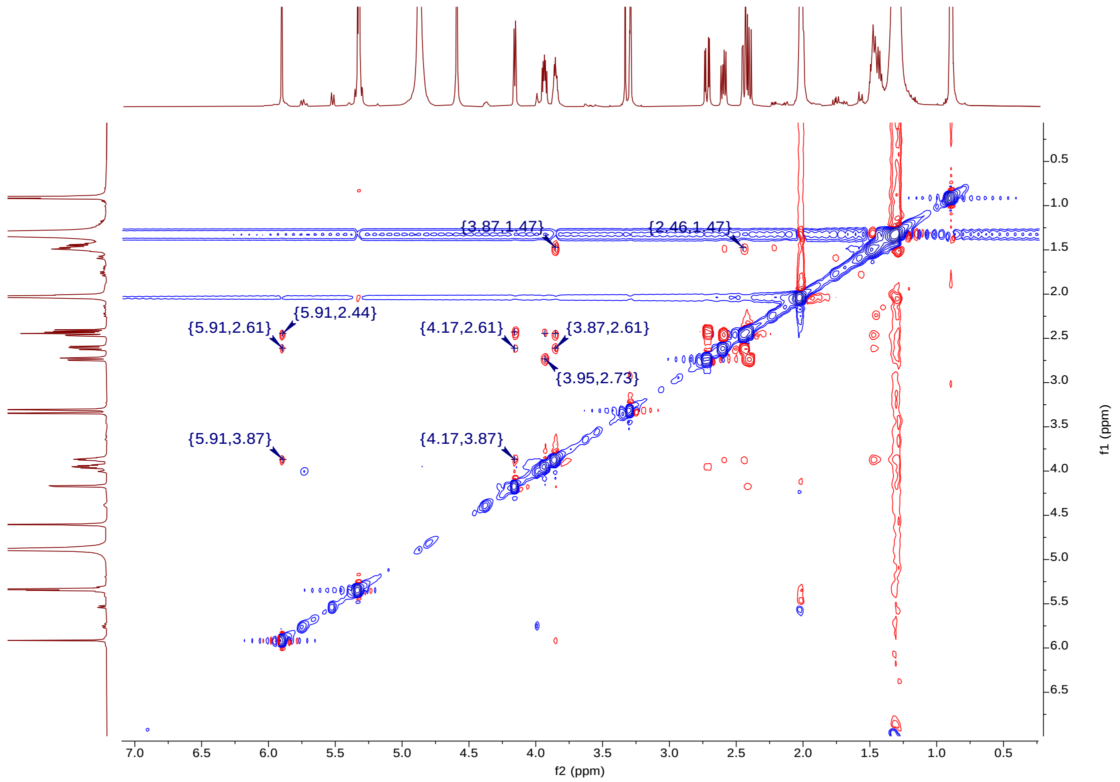

Figure S5.7. HRESIMS spectrum of compound **5**.

Qualitative Analysis Report

**Data Filename**	PQ-6523-.d	**Sample Name**	PQ-6523
**Sample Type**	Sample	**Position**	P1-F7
**Instrument Name**	Instrument 1	**User Name**	
**Acq Method**	s-.m	**Acquired Time**	11/20/2023 11:51:38 AM
**IRM Calibration Status**	Success	**DA Method**	PCDL.m
**Comment**			

Sample Group **Info.**

Acquisition SW Version 6200 series TOF/6500 series Q-TOF B.05.01 (B5125.2)

User Spectra

Fragmentor Voltage	Collision Energy	Ionization Mode
135	0	ESI

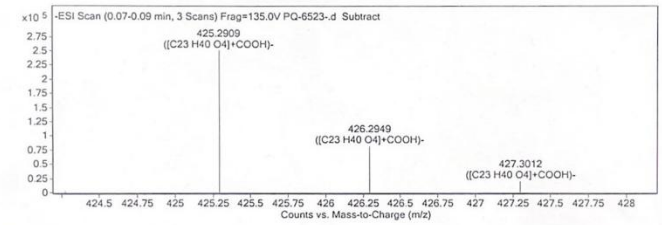

Peak List

m/z	z	Abund	Formula	Ion
112.9854	1	20630.8		
425.2909	1	251324.2	C23 H40 O4	(M+COOH)-
426.2949	1	82465.37	C23 H40 O4	(M+COOH)-
442.2812	1	157223.42		
443.285	1	56265.94		
493.2783	1	220043.44		
494.2821	1	75882.94		
759.5783	1	100310.25		
760.5822	1	69461.89		
761.5861	1	24787.55		

Formula Calculator Element Limits

Element	Min	Max
C	3	60
H	0	150
O	0	30

Formula Calculator Results

Formula	CalculatedMass	CalculatedMz	Mz	Diff. (mDa)	Diff. (ppm)	DBE
C23 H40 O4	380.2927	425.2909	425.2909	0.00	0.00	4.0000

--- End Of Report ---

Agilent Technologies Page 1 of 1 Printed at: 11:52 AM on: 11/20/2023

Figure S5.8. UV spectrum of compound **5** in MeOH.

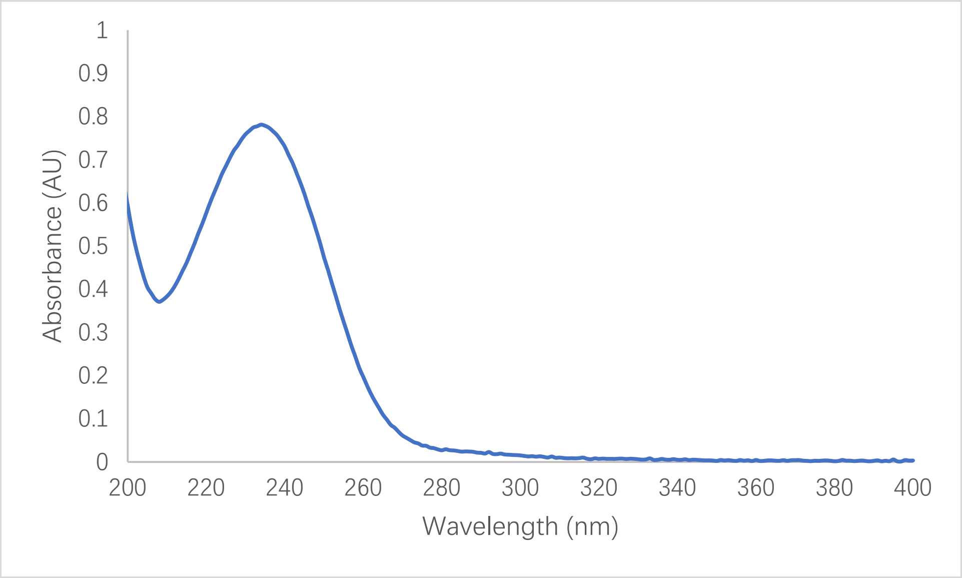

Figure S5.9. IR spectrum of compound **5** in KBr disk.

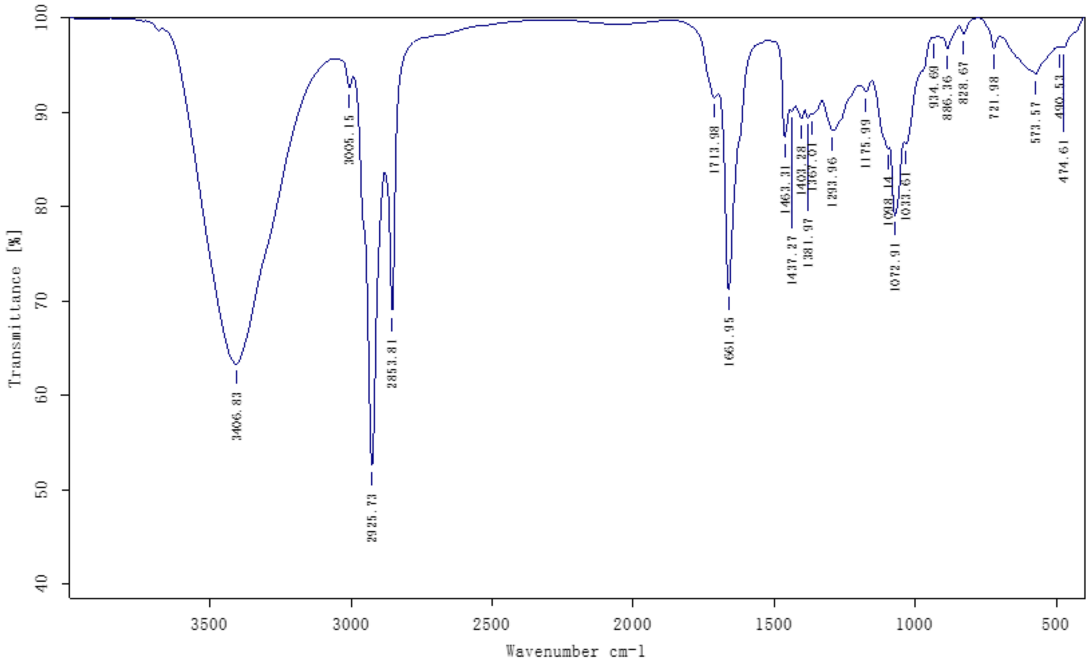

Figure S6.1. ^{1}H NMR spectrum of compound **6** in CD_3OD.

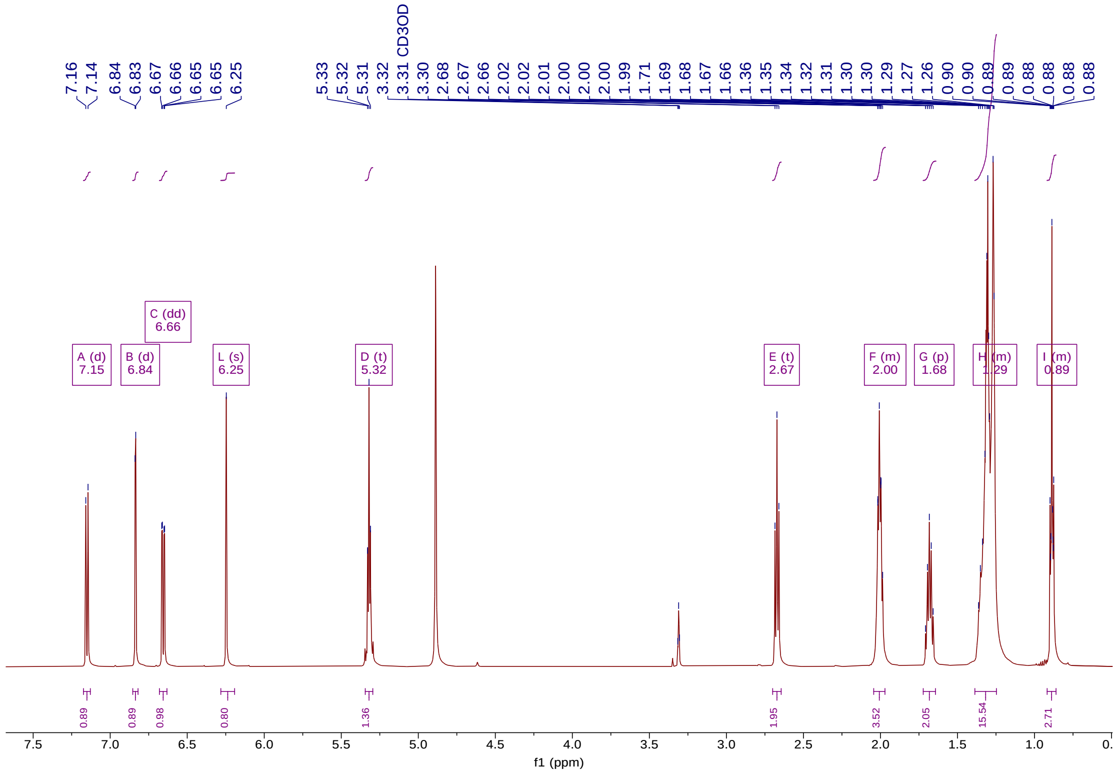

Figure S6.2. ^{13}C NMR and DEPT spectrum of compound **6** in CD_3OD.

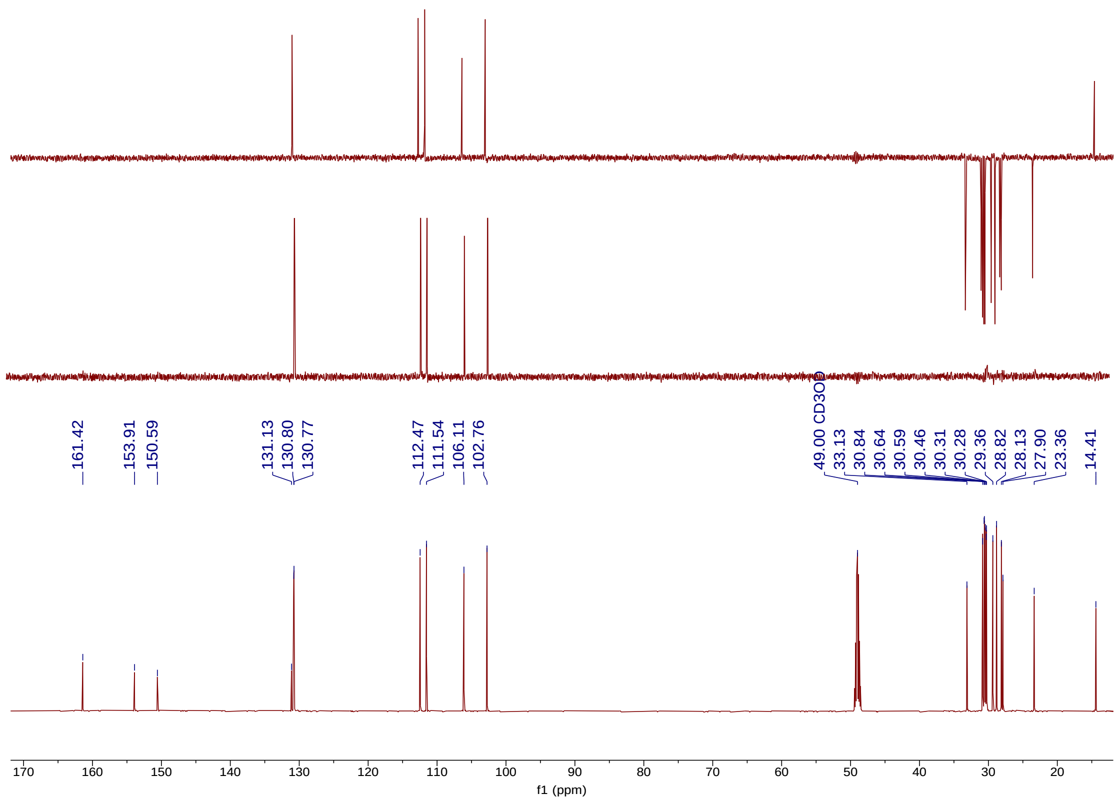

Figure S6.3. ^{1}H-^{1}H COSY spectrum of compound **6** in CD_3OD.

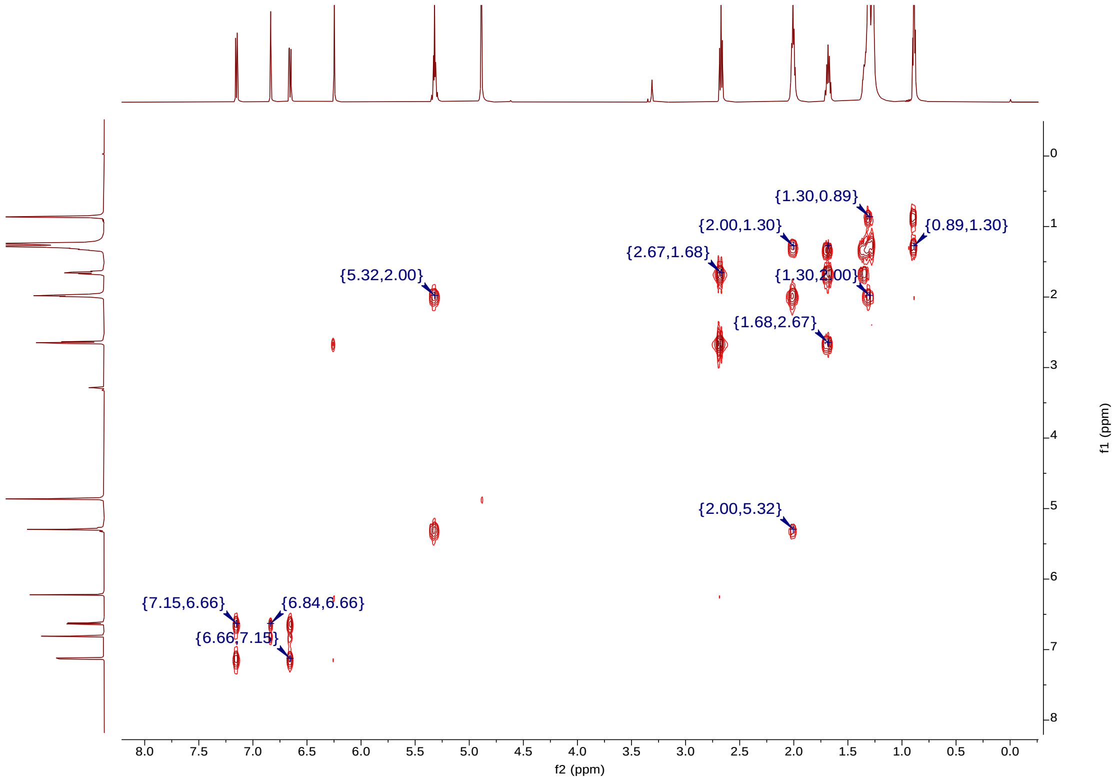

Figure S6.4. HSQC spectrum of compound **6** in CD_3OD.

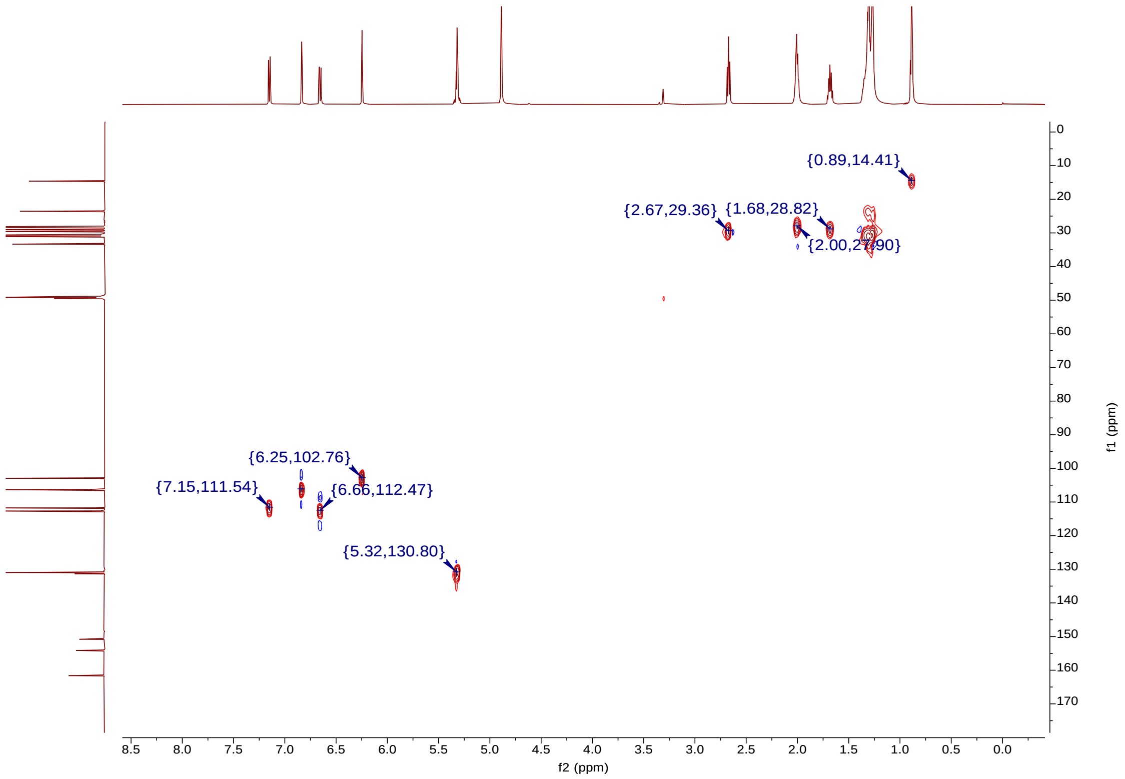

Figure S6.5. HMBC spectrum of compound **6** in CD_3OD.

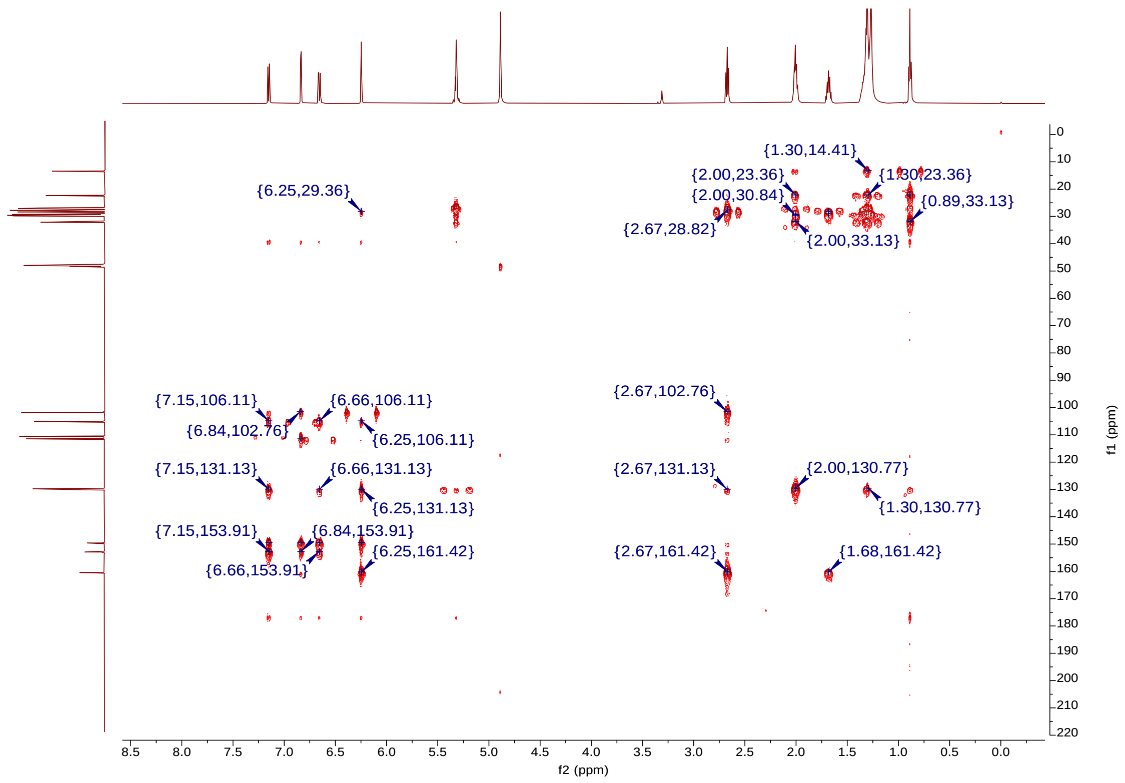

Figure S6.6. HRESIMS spectrum of compound **6**.

Qualitative Analysis Report

**Data Filename**	PQ-6441.d	**Sample Name**	PQ-6441
**Sample Type**	Sample	**Position**	P1-F8
**Instrument Name**	Instrument 1	**User Name**	
**Acq Method**	s-.m	**Acquired Time**	11/20/2023 11:43:10 AM
**IRM Calibration Status**	Success	**DA Method**	PCDL.m
**Comment**			

Sample Group **Info.**

Acquisition SW Version 6200 series TOF/6500 series Q-TOF B.05.01 (B5125.2)

User Spectra

Fragmentor Voltage	Collision Energy	Ionization Mode
135	0	ESI

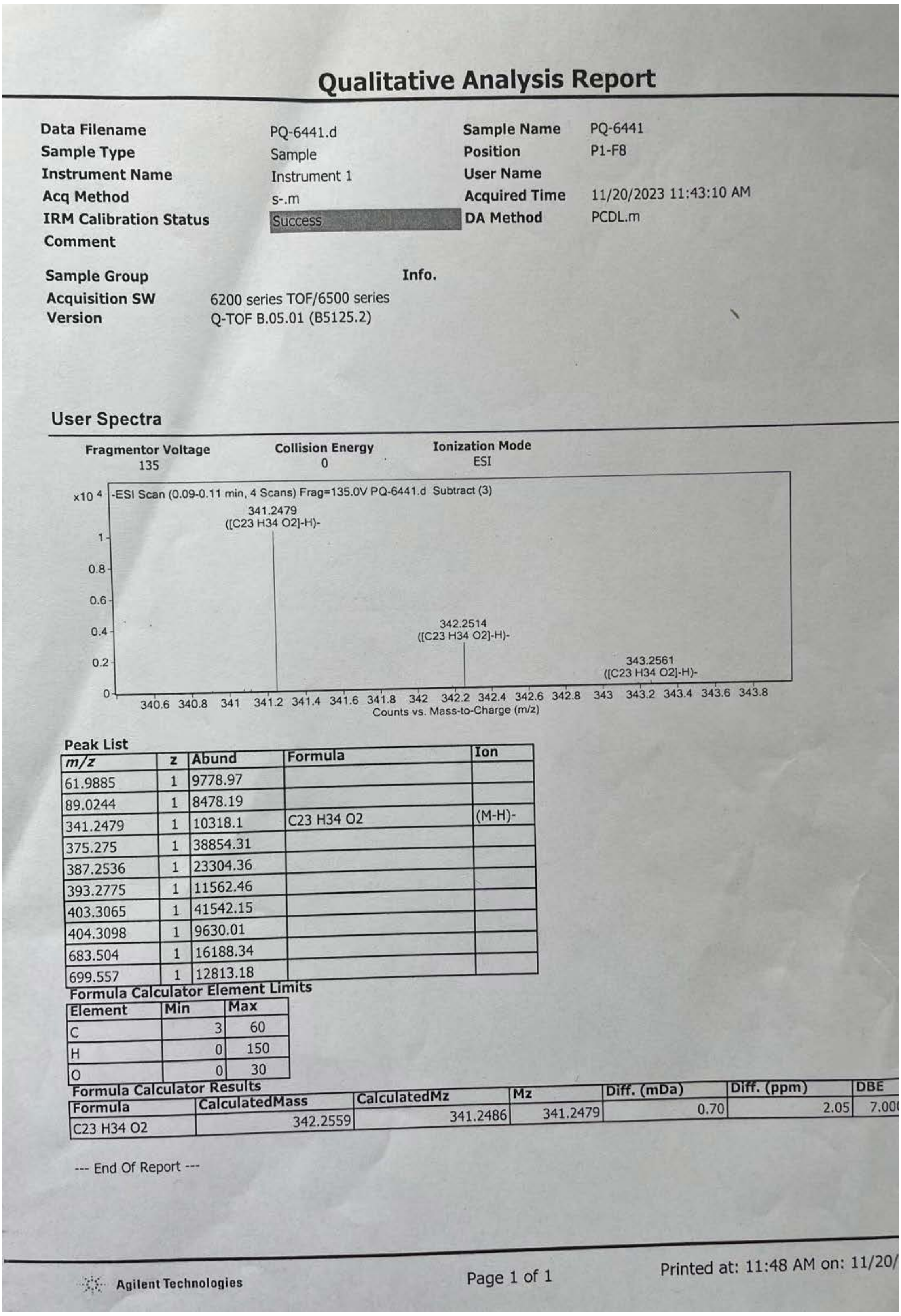

Peak List

m/z	z	Abund	Formula	Ion
61.9885	1	9778.97		
89.0244	1	8478.19		
341.2479	1	10318.1	C23 H34 O2	(M-H)-
375.275	1	38854.31		
387.2536	1	23304.36		
393.2775	1	11562.46		
403.3065	1	41542.15		
404.3098	1	9630.01		
683.504	1	16188.34		
699.557	1	12813.18		

Formula Calculator Element Limits

Element	Min	Max
C	3	60
H	0	150
O	0	30

Formula Calculator Results

Formula	CalculatedMass	CalculatedMz	Mz	Diff. (mDa)	Diff. (ppm)	DBE
C23 H34 O2	342.2559	341.2486	341.2479	0.70	2.05	7.00

--- End Of Report ---

Agilent Technologies Page 1 of 1 Printed at: 11:48 AM on: 11/20/

Figure S6.7. UV spectrum of compound **6** in MeOH.

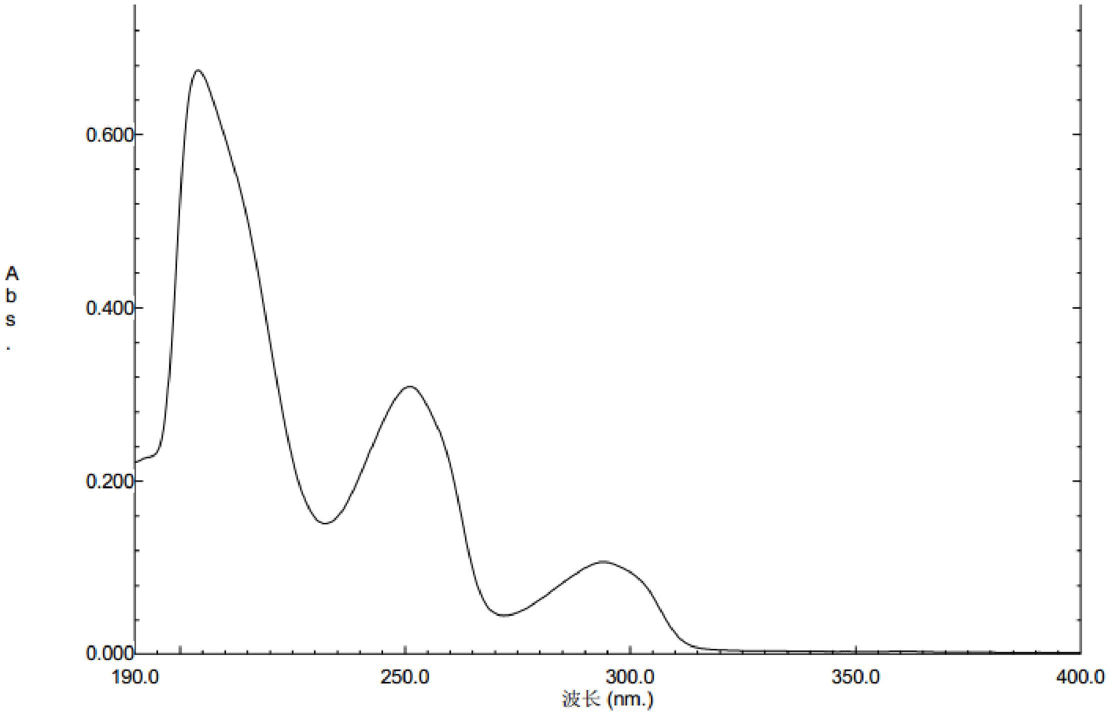

Figure S6.8. IR spectrum of compound **6** in KBr disk.

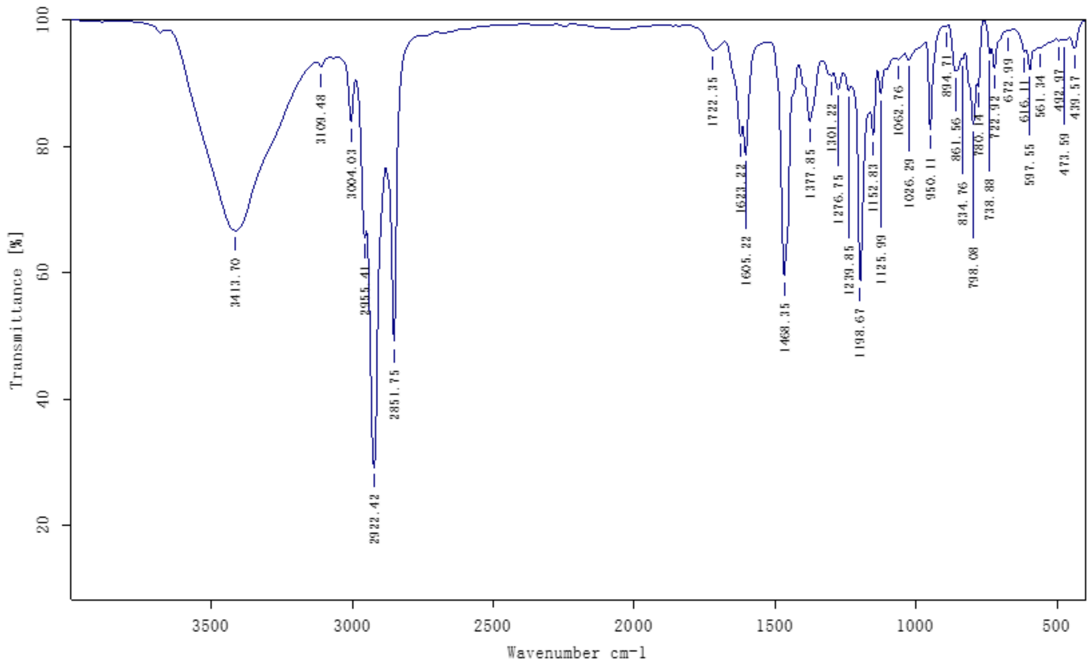

Figure S7.1. ^{1}H NMR spectrum of compound **7** in CD_3OD.

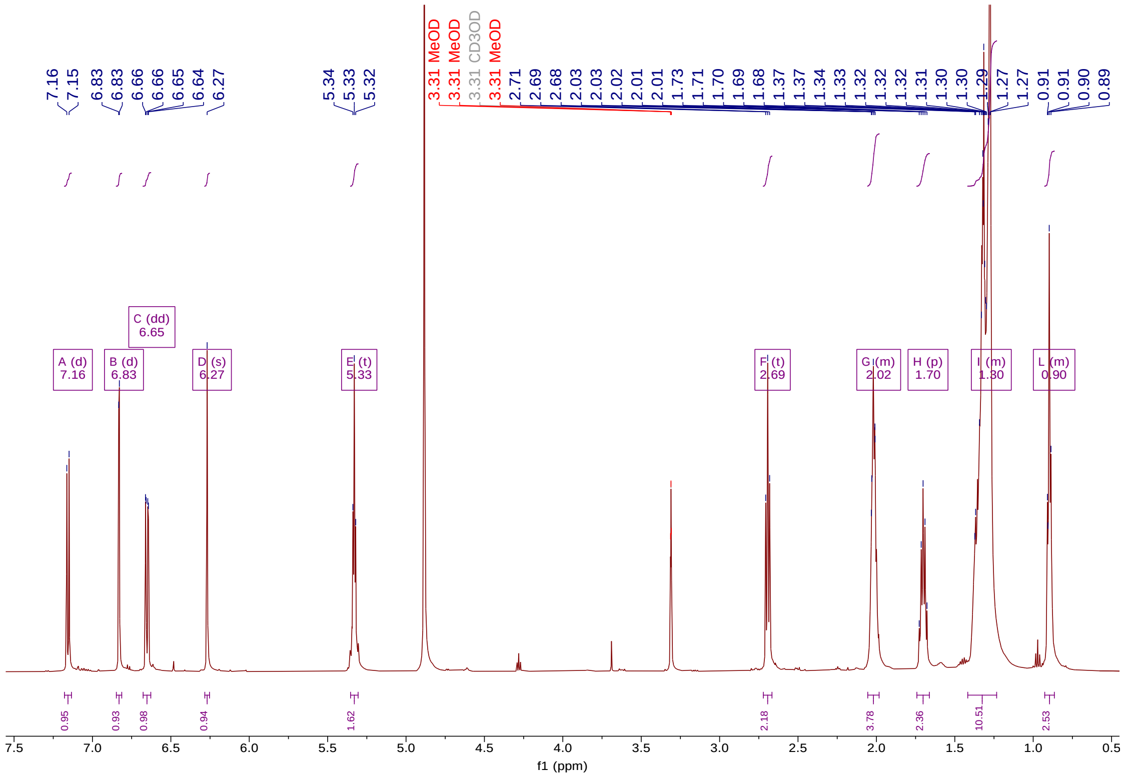

Figure S7.2. ^{13}C NMR and DEPT spectrum of compound **7** in CD_3OD.

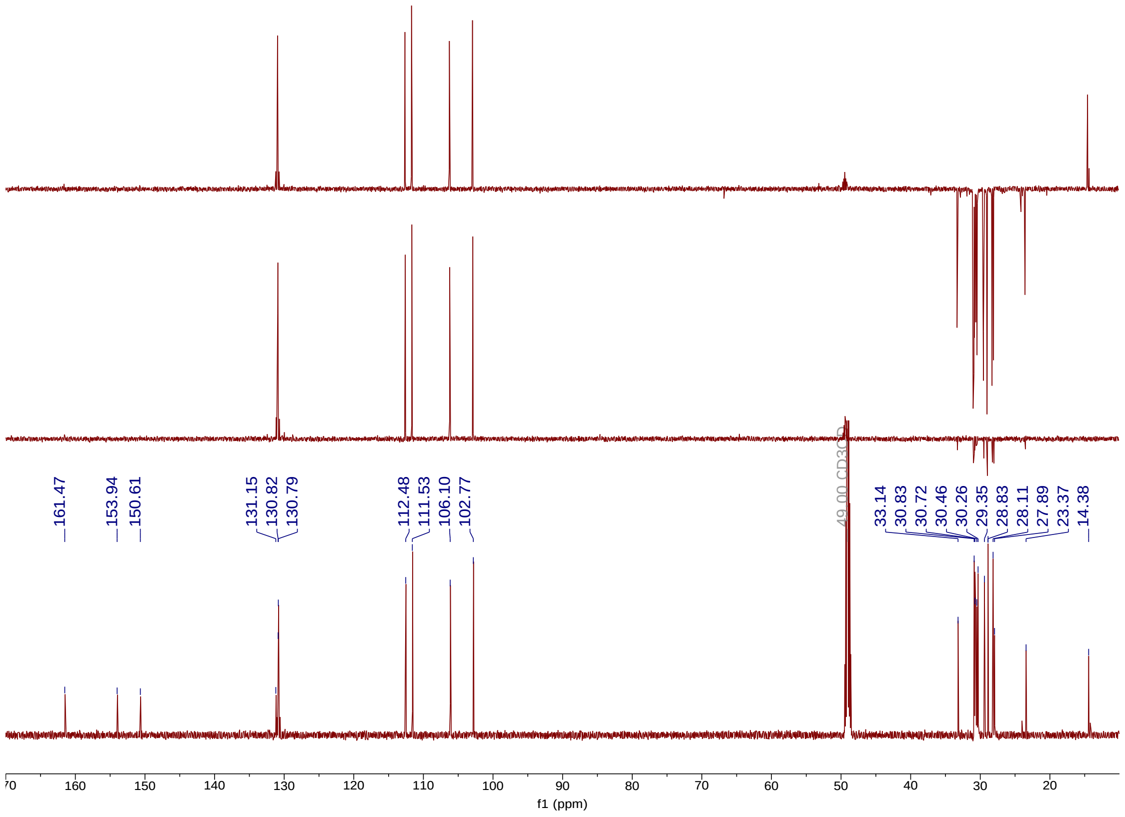

Figure S7.3. ^{1}H-^{1}H COSY spectrum of compound **7** in CD_3OD.

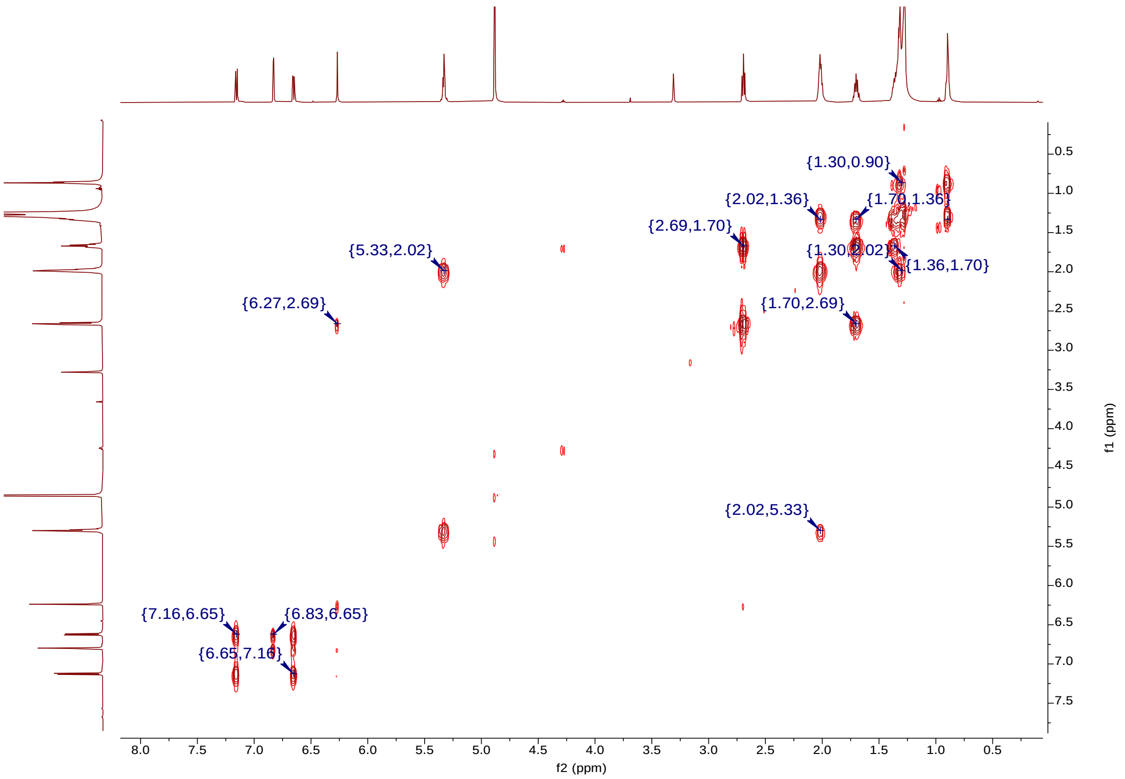

Figure S7.4. HSQC spectrum of compound **7** in CD_3OD.

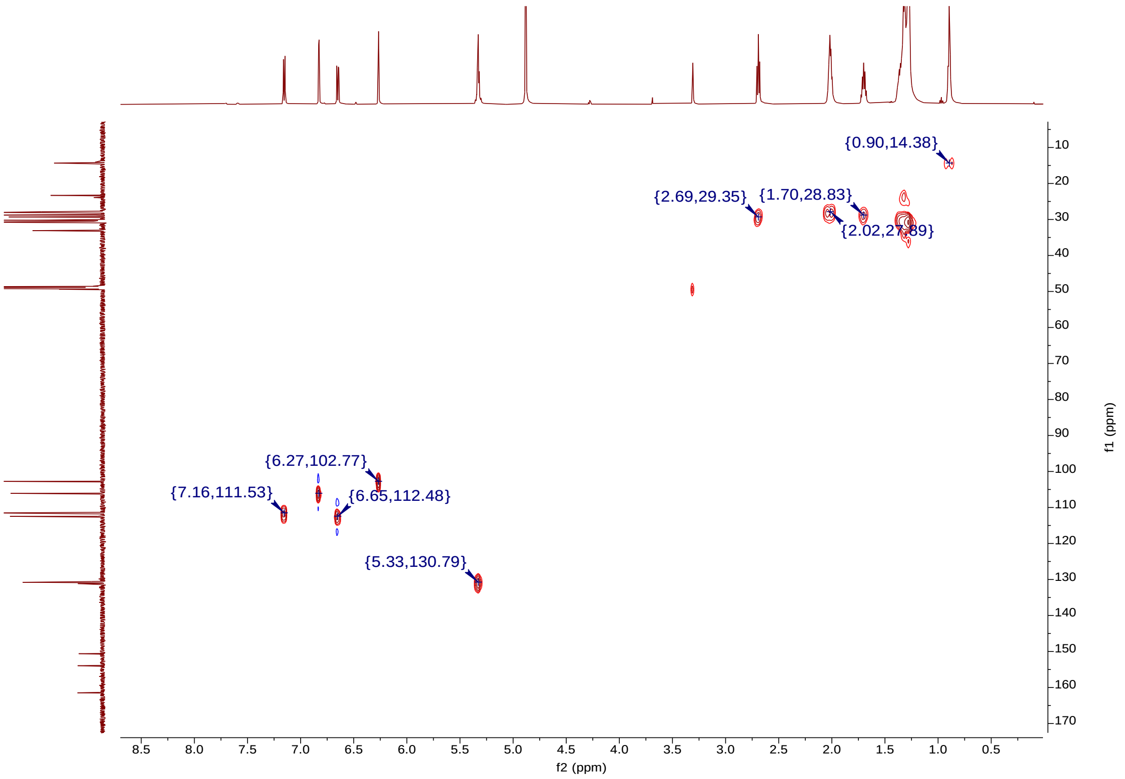

Figure S7.5. HMBC spectrum of compound **7** in CD_3OD.

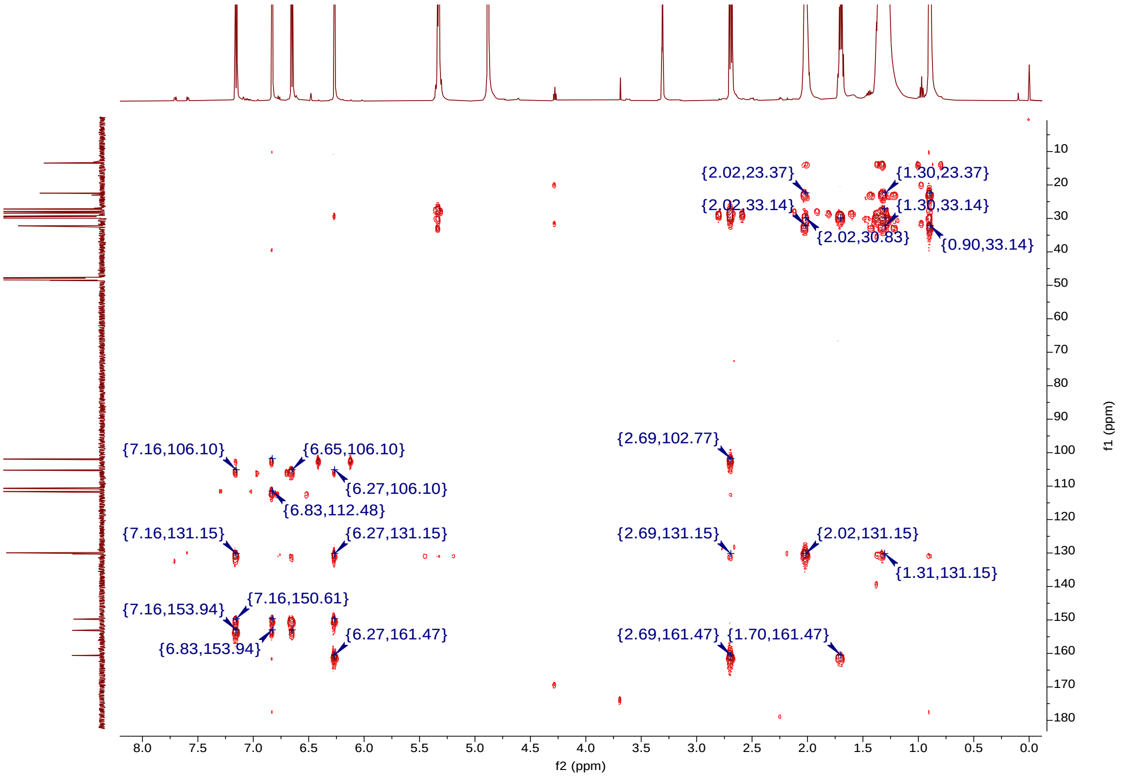

Figure S7.6. HRESIMS spectrum of compound **7**.

Qualitative Analysis Report

**Data Filename**	644113.d	**Sample Name**	644113
**Sample Type**	Sample	**Position**	P1-F4
**Instrument Name**	Instrument 1	**User Name**	
**Acq Method**	s.m	**Acquired Time**	2/4/2024 4:32:43 PM
**IRM Calibration Status**	Success	**DA Method**	PCDL.m
**Comment**			

Sample Group **Info.**

Acquisition SW Version 6200 series TOF/6500 series Q-TOF B.05.01 (B5125.2)

User Spectra

Fragmentor Voltage	Collision Energy	Ionization Mode
165	30	ESI

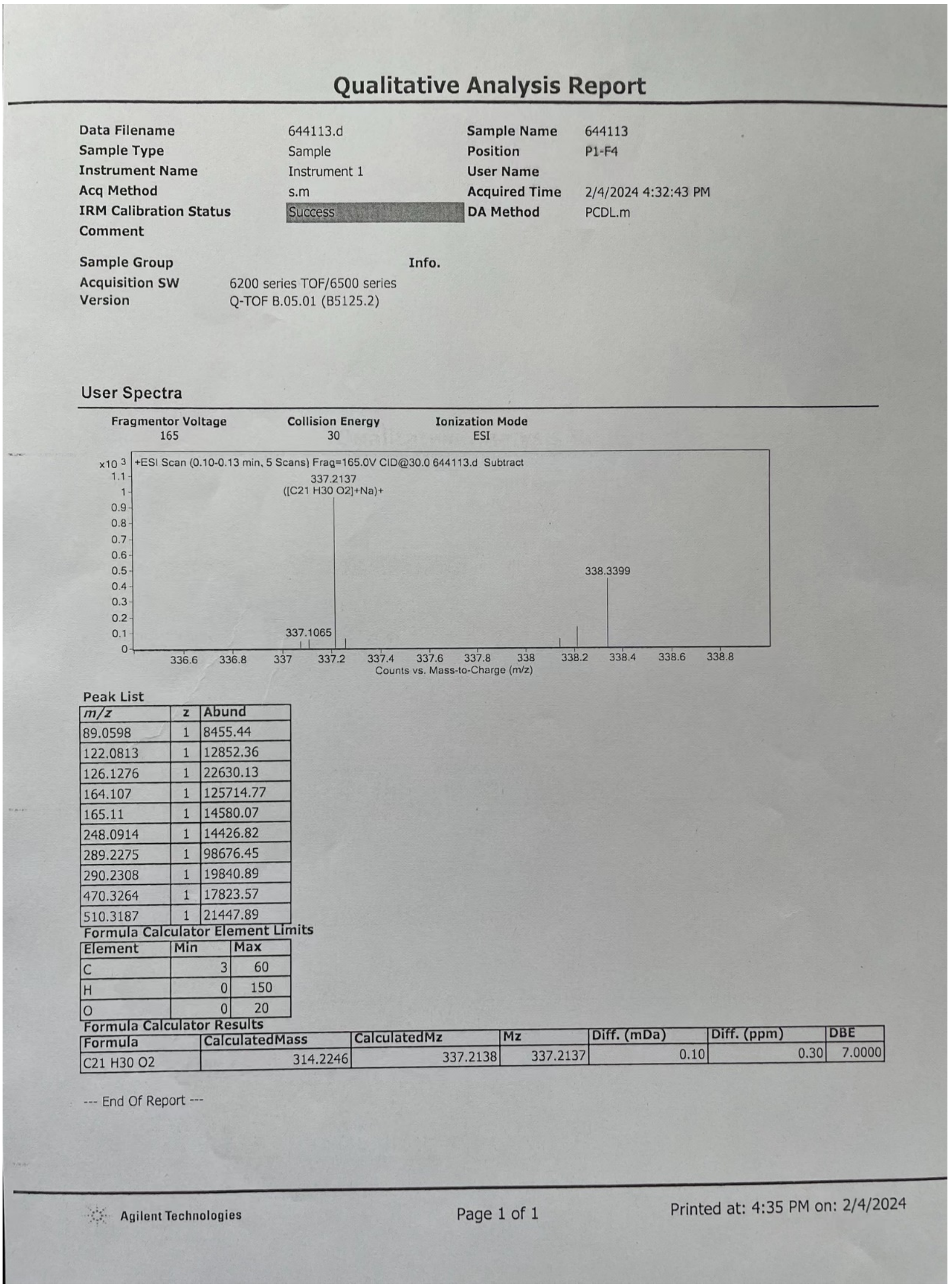

Peak List

m/z	z	Abund
89.0598	1	8455.44
122.0813	1	12852.36
126.1276	1	22630.13
164.107	1	125714.77
165.11	1	14580.07
248.0914	1	14426.82
289.2275	1	98676.45
290.2308	1	19840.89
470.3264	1	17823.57
510.3187	1	21447.89

Formula Calculator Element Limits

Element	Min	Max
C	3	60
H	0	150
O	0	20

Formula Calculator Results

Formula	CalculatedMass	CalculatedMz	Mz	Diff. (mDa)	Diff. (ppm)	DBE
C21 H30 O2	314.2246	337.2138	337.2137	0.10	0.30	7.0000

--- End Of Report ---

Agilent Technologies Page 1 of 1 Printed at: 4:35 PM on: 2/4/2024

Figure S7.7. UV spectrum of compound **7** in MeOH.

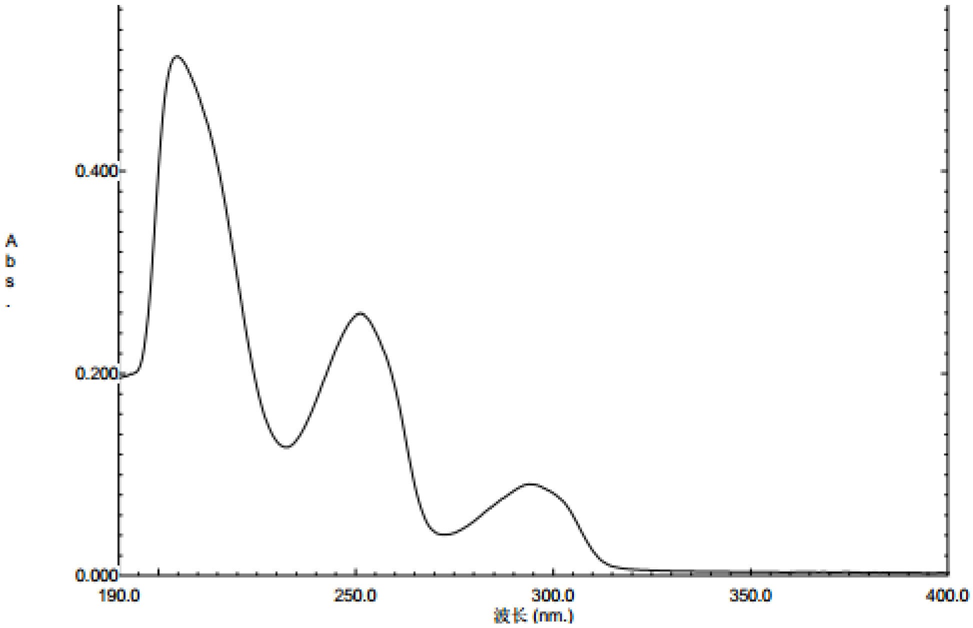

Figure S7.8. IR spectrum of compound **7** in KBr disk.

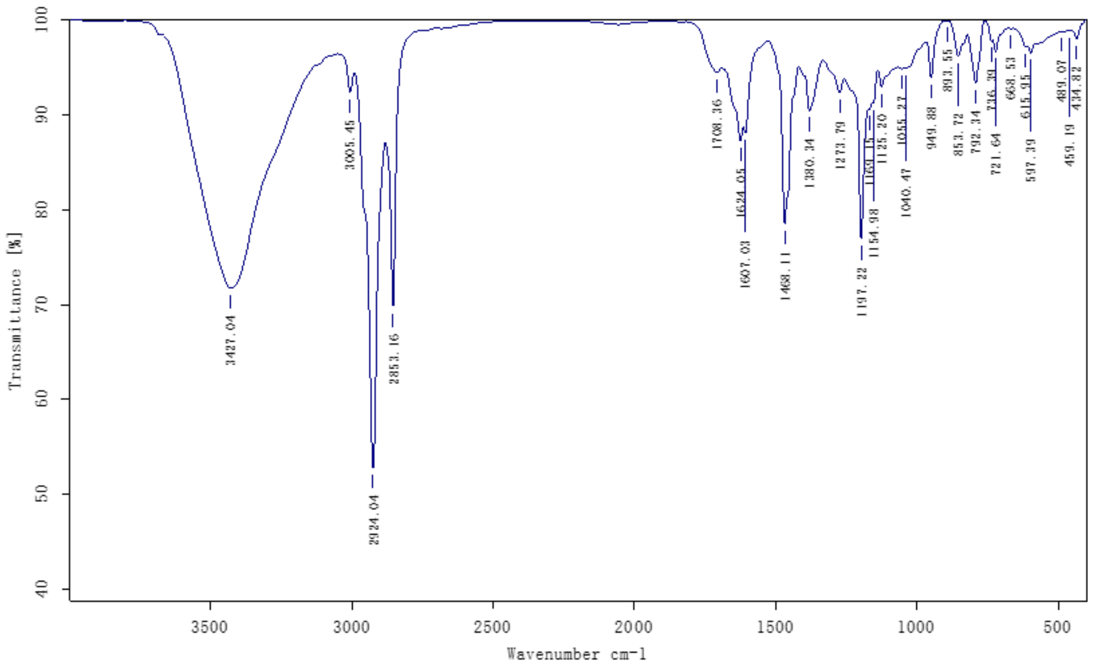

Figure S8.1. ^{1}H NMR spectrum of compound **8** in CD_3OD.

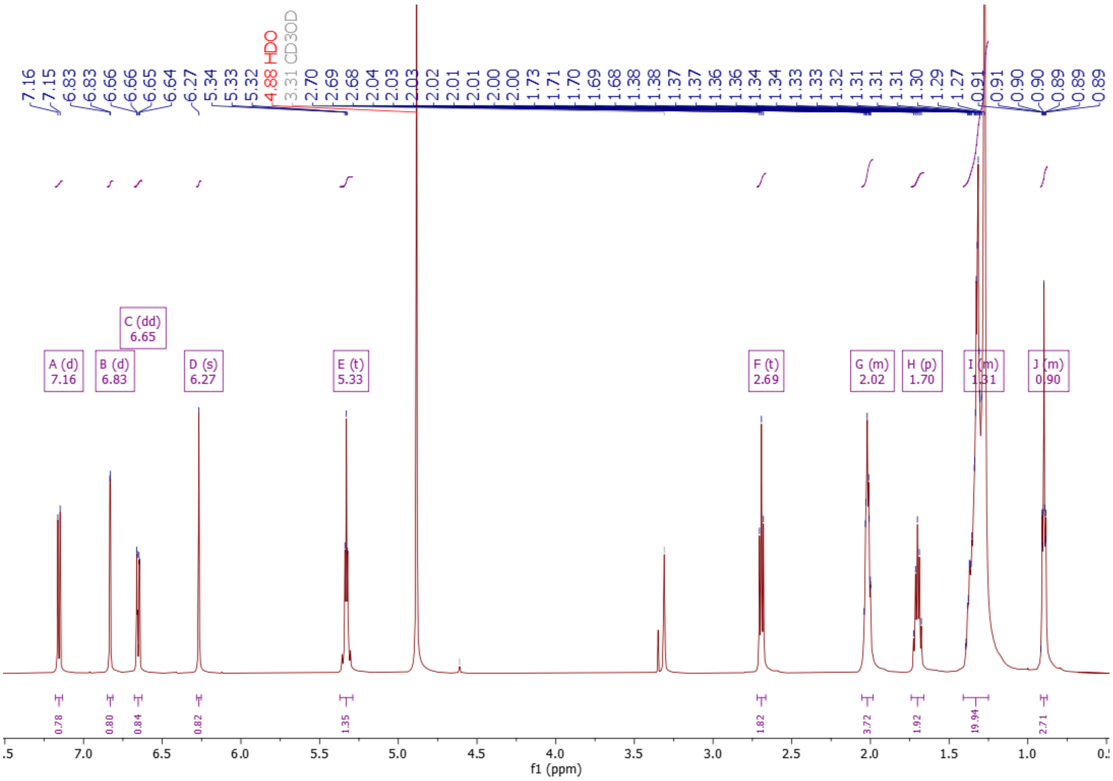

Figure S8.2. ^{13}C NMR and DEPT spectrum of compound **8** in CD_3OD.

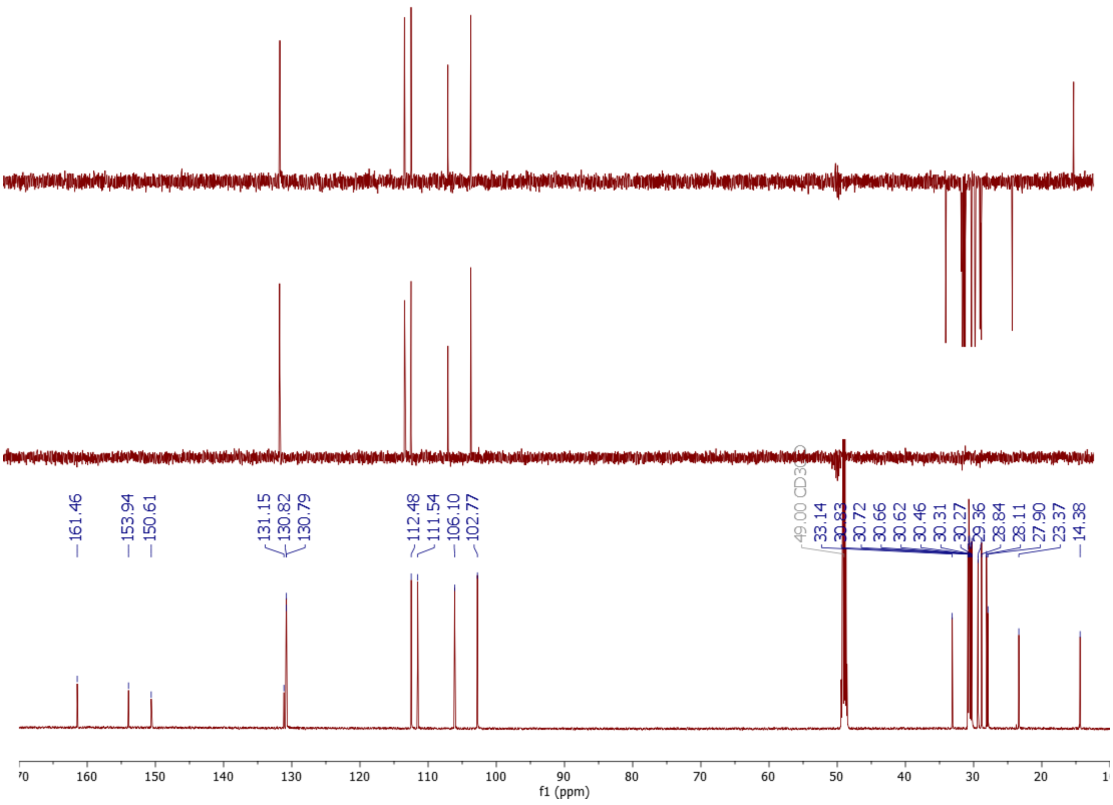

Figure S8.3. HSQC spectrum of compound **8** in CD_3OD.

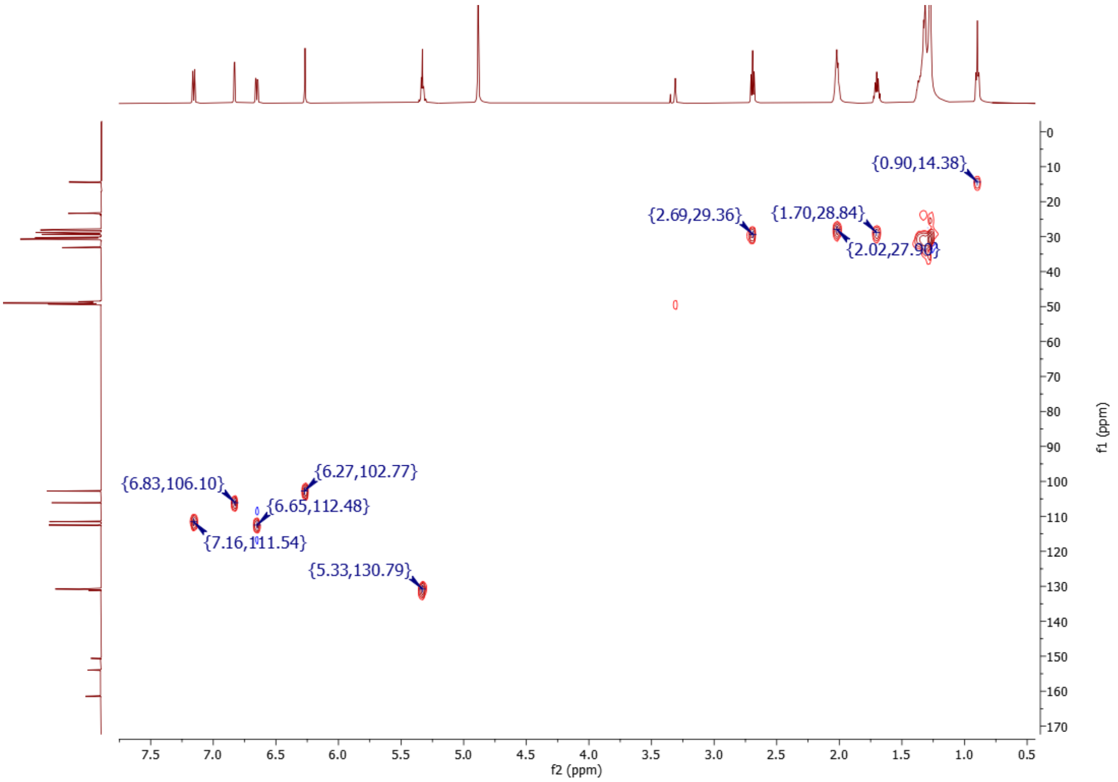

Figure S8.4. HMBC spectrum of compound **8** in CD_3OD.

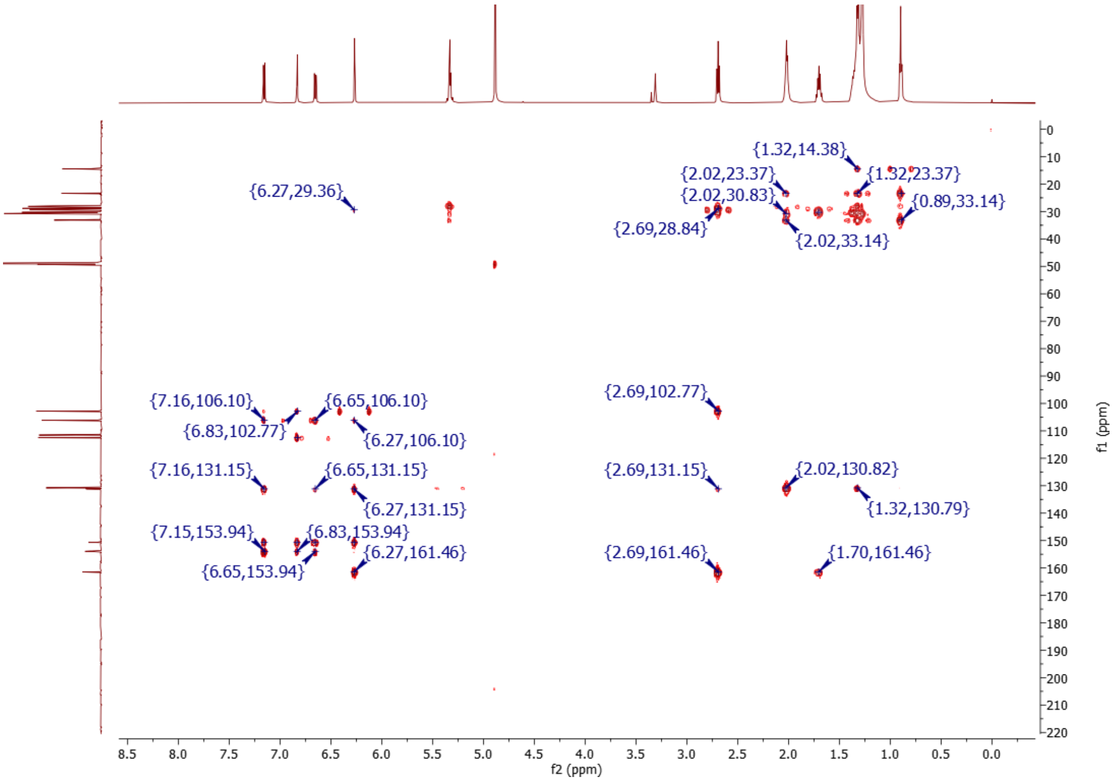

Supplement: Supplementary file 1 [file foods-13-01495-s001.zip › foods-2974344-supplementary.pdf]
